# Supplementary material for: De novo design of an intercellular signaling toolbox for multi-channel cell–cell communication and biological computation
Source: Nat Commun. 2020 Aug 24;11:4226. doi: 10.1038/s41467-020-17993-w (PMC7445162; doi:10.1038/s41467-020-17993-w)
Supplement: Supplementary file 1 — Supplementary Information [file 41467_2020_17993_MOESM1_ESM.pdf]

Supplementary Information

De novo **design of an intercellular signaling toolbox for multi-channel cell-cell communication and biological computation**

Pei Du et. al

Supplementary Methods

Design of the responsive promoters for IV, pC and MMF systems

IV system: The putative *bjaI* promoter from *B. Japonicum* was extracted and found irresponsive to the IV signal. Moreover, it was not clear whether BjaR functions as an activator or as a repressor, although the BjaR operator shares similar inverted repeat sequences with the LuxR operator[1]. Therefore, we designed a LuxR-type promoter (P_{bja*}) by inserting the BjaR operator into the P_{lux*} promoter scaffold as well as an EsaR-type promoter ($P_{bja\text{-}mut2}$) by placing two BjaR operators at the *core* and the *proximal* positions in case BjaR functions as a repressor (Supplementary Figure 1). Our results showed that only the receiver with the P_{bja*} promoter responded to the IV signal (Figure 2a; Supplementary Figure 1). Chemically synthesized IV-HSL was used for measuring the induction curve (Supplementary Figure 21).

pC system: Like BjaI, the putative RpaI promoter extracted from *R. Palustris* did not respond to the pC signal (Supplementary Figure 1). Thus, based on published RpaR consensus binding sequence[2], we designed two activator-type synthetic promoters. As a LuxR-type transcription activator, RpaR may function in the same way as LuxR does. So we designed a synthetic promoter (P_{rpa*}) by the same strategy as that of designing P_{bja*}. Alternatively, the putative RpaI promoter might have failed to function in *E. coli* due to incompatibility of its -10 region. Therefore, a second promoter ($P_{rpa\text{-}mut2}$) was designed by mutating the original -10 region of the putative RpaI promoter to TACTTT while keeping the rest of promoter intact (Supplementary Figure 1). Our results showed that only the receiver with the first synthetic promoter (P_{rpa*}) can be activated by the pC signal (Figure 2a; Supplementary Figure 1).

MMF system: The MmfR regulator is a TetR-family repressor which recognizes known MmfR operator sequences[3]. We designed 12 operators by randomizing two degenerate positions in the MmfR operator sequence and inserted each of them in the P_{tac} promoter, resulting in 12 different promoters (Supplementary Figure 2). Upon expression of MmfR, the 12 constructed P_{mmfR} promoters show highly diverse response, with dynamic ranges from 0 to 33 folds of decrease in fluorescence (Supplementary Figure 2). The promoter (P_{mmfR5}) with 33-fold repression by MmfR was selected and renamed P_{mmf}. We verified that the receiver cells with P_{mmf} were activated by the MMF signal (Figure 2a; Supplementary Figure 2).

Optimization of the well-studied quorum sensing systems

C8 system: The native *cepI* promoter from *B. cepacia* has been reported to function in *E. coli*[4], but shows very small dynamic range to respond C8-HSL signal (Supplementary Figure 3b). To resolve this issue, we redesigned the responsive promoter by combining the CepO with the core region of P_{lux*} promoter to result in five P_{lux*}-like promoters (Supplementary Figure 3a). One of the engineered promoters ($P_{cep\text{-}mut4}$) increased the maximum output and dynamic range comparing to the native

promoter. The chosen $P_{cep\text{-}mut4}$ promoter was subsequently re-named as P_{cep*}. (Supplementary Figure 3b).

3OC6 system: The 3OC6 inducible P_{lux} promoter of *V. fischeri* had been optimized by mutating the -10 region from TAGAGT to TACTTT[5], and the resulting P_{lux*} promoter exhibited higher dynamic range comparing to the native P_{lux} promoter, possible due to the reduced background expression[5]. Our test results show the 3OC6 receiver cells can be activated by its cognate sender cells (Supplementary Figure 4).

C4 system: The previously published C4 inducible promoter ($P_{rhl*\text{-}catLVA}$) was designed by inserting a *P. aeruginosa* origin rhlA operator for RhlR[6] into the P_{lux} promoter and replace the LuxR operator. We further reduce the basal expression of the synthetic promoter (P_{rhl*}) by changing the -10 region of $P_{rhl*\text{-}catLVA}$ promoter to TACTTT. Experimental results show P_{rhl*} receiver responds to its cognate sender (Supplementary Figure 4).

3OC12 system: We also inserted the LasI operator into the P_{lux} promoter resulting in a new P_{las*} promoter (Supplementary Figure 4). Our results show that the receiver cells containing P_{las*} promoter can be activated by the 3OC12 sender (Supplementary Figure 4).

Characterization of *de novo* designed cell-cell communication systems

Sal system: The Sal receiver part was constructed with the native promoters (P_{sal}) from *P. putida*, which has been reported to function in *E. coli*[7]. The receiver with P_{sal} promoter can be activated by the Sal sender containing either *pchBA* or *irp9* genes (Figure 2a; Supplementary Figure 6).

DAPG system: The DAPG receiver part was constructed with P_{phlF} promoter which was initially designed for the screening of TetR family repressors in previous publication[8]. A C1434 oligomerization domain was fused to PhlF, creating a PhlF-C1434 fusion protein to take advantage of the cooperative regulatory capability from C1434 dimerization[9]. The sender of DAPG was constructed with the *phlACBD* cluster extracted from *P. luminescence* and heterologously expressed in *E. coli* (Figure 1). Our results showed that the DAPG receiver can be activated by its sender (Figure 2a; Supplementary Figure 6).

NG system: The NG receiver was constructed with the native P_{fdeA} promoter which has been reported to function in *E. coli*[10]. This receiver could respond to 1mM NG induction with 165 folds increase of fluorescence (Supplementary Figure 7). Like pC signal molecule, NG synthesis requires the same p-coumaroyl-CoA synthesized by TAL and 4CL from tyrosine metabolism. Our NG sender part also contains additional *chs* and *chi* genes from higher plant *Petunia X hybrid* and *Medicago sativa*, in order to utilize Malonyl-CoA as the other precursor for NG synthesis[11] (Figure 1). By co-culturing with the sender cell, our results show that the NG receiver can be successfully activated (Figure 2a; Supplementary Figure 6).

Pyrone systems: The pyrone receiver was constructed with the native P_{pcfA} promoters from *P. luminescens*[12]. The Pyrone sender was constructed with *bkdABC* cluster, *ngrA* and *ppyS* genes extracted from *P. luminescens* (Figure 1). The pyrone receiver cells failed to respond to its sender cells.

Uric acid system: To construct the receiver of Uric acid, we adopted a published P_{hucR} promoter which was designed by inserting the HucR operator of *D. radiodurans* between the -35 and -10 regions of Ptac promoter[13]. The P_{hucR} receiver can be activated upon induction by 0.2mM uric acid (Supplementary Figure 7). Unfortunately, we were unable to construct a functional sender cell of uric acid to activate the uric acid receiver cell.

A-factor and SCB1 systems: The ArpA and ScbR are also TetR family repressors with published operator sequences[14, 15]. Therefore, we designed several different promoters by combining the operators with P_{tac} or J23119 promoters. However, our designs of promoters with both ArpA and ScbR operators was unable to be repressed by their cognate repressors. We were thus unable to construct functional receiver for the A-factor and SCB1.

Quantitation of sensitivity of artificial cell-cell communication systems

To determine the dynamic range and sensitivity of each cell-cell communication system, the curve of each sender-receiver response can be fitted with a simple Hill function

$$y = f(x) = y_{\min} + (y_{\max} - y_{\min})\frac{x^n}{K^n + x^n} \qquad (1)$$

where y is the receiver's output fluorescence; $y_{\min}$ is the minimal output; $y_{\max}$ is the maximal output; K is the concentration of signaling molecule where output fluorescence is 50% of the maximal value, named EC50; and n is the Hill coefficient. Therefore, the dynamic range of each curve can be calculated by $y_{\max}/y_{\min}$. To characterize the sensitivity of each system, the sender-receiver response curves were fitted to Supplementary Equation 1 and calculated the EC50 values, as well as the EC10 and EC90 which are the concentration of the signaling molecule where output fluorescence are 10% and 90% of the maximal value.

Transferring cell-cell communication systems from *E. coli* into mammalian and *S. cerevisiae* cells

To transfer the *de novo* designed intercellular communication system from one host to another, appropriate modifications of their regulatory elements must be made for sender and receiver parts in order to be functional in new hosts. For example, the promoters that expresses the biosynthetic and regulatory genes must be modified to transcribe and translate them in the new hosts. Especially, transferring them from *E. coli* into human and other eukaryotic cell lines, their regulatory promoters must be completely redesigned, because the mechanisms of transcription initiation in eukaryotic cells are significantly different.

Homo sapiens**:** To transfer the pC cell-cell communication system from *E. coli* into human cells, we constructed a sender and a receiver using the human embryonic kidney cells line, 293T cell line, as host. To construct the pC sender, the three biosynthetic genes (*tal*, *4cl* and *rpaI*) were inserted into lentiviral vectors together with reporter genes, in order to create stable cell line through lentiviral infection and flow cytometric sorting. The *tal*, *rpaI* genes were inserted into a lentiviral vector together with a constitutive expressed *iRFP* reporter gene, connected by P2A and T2A peptides. The *4Cl* gene was inserted into another lentiviral vector together with a constitutive expressed *mTurquoise2* reporter gene, connected by a P2A peptide. Both lentiviral vectors contain a constitutive pEF1α promoter to express all the inserted genes. Stable sender cell line was created after co-infection with the two types of lentivirus and flow cytometric sorting for the double positive (iRFP+ and mTourquoise2+) population. To construct the pC receiver cell lines, we first fused the allosteric TF RpaR with a VTR3 activation domain to create a new eukaryotic transcriptional activator[16]. Such VTR3-RpaR design enables binding to RpaR operator and sense pC-HSL signal. Then, we designed CMV1 and CMV3G promoters by inserting two RpaR operator upstream of a miniCMV[17] or a TRE3G core promoter, respectively, reported by yellow fluorescent protein (Citrine) (Supplementary Figure 11a). In addition, a red fluorescent protein (mCherry) was constitutively expressed on the same plasmid as internal control of the plasmid transfections (Supplementary Figure 11d).

To examine the cell-cell communication in 293T cells, the media of stable sender cell line was harvested after 48h of culture, while the receiver plasmid was transiently transfected into 293T cells and mixed with equal volume of freshly harvested sender media 3h after transfection. Cells were subsequently cultured for 48h before being harvested into PBS buffer and flow cytometric analysis for yellow and red fluorescence. Our data show that the 293T receiver cells with either CMV1 or CMV3G promoter can be activated by the pC-HSL signaling molecule synthesized by the 293T sender cell (Figure 2b; Supplementary Figure 11b).

S. cerevisiae: To transfer cell-cell communication systems to *S. cerevisiae*, we designed a DAPG responsive promoter by combining the PhlF operator with the *S. cerevisiae* P_{Gal} promoter. The P_{Gal} promoter is known to drive gene expression on both upstream and downstream direction with P_{Gal10} and P_{Gal1} promoters, respectively. So we designed a $P_{phlF\text{-}Y}$ promoter by inserting two PhlF operators between the TATA box and transcription start site of P_{Gal1} promoter (Figure 2b; Supplementary Figure 13). Meanwhile, a PhlF-C1434 fusion protein was expressed by the P_{Gal10} promoter to create ultrasensitive response. Our results show that, with 2% galactose in culture medium, the promoter activity is tightly repressed by PhlF-C1434 before DAPG induction. The *S. cerevisiae* receiver can be activated with chemically and biological synthetic DAPG molecule (Figure 2b; Supplementary Figure 13b).

Besides the DAPG system, we also achieved cross-kingdom communication with the Sal system. The *irp9* gene or the *pchBA* cluster was inserted into the genome of *S. cerevisiae* under the control of a P_{tet} promoter (Supplementary Figure 11a). Upon

induction by aTc, the two *S. cerevisiae* strains synthesize Sal. Our data show that the *E. coli* receiver can be activated with the medium of both the *pchBA* strain and the *irp9* strain (Supplementary Figure 13c).

Quantitation of mammalian cell-cell communication

In *E. coli* and yeast, the output fluorescence of each sample was defined as the average value of the fluorescence intensity from all the single cells within the sample. In mammalian cells, the output fluorescence had to be calculated using a different method for the following reason.

To construct the mammalian receiver strain, the receiver plasmid was transiently transfected into HEK293T cells prior to induction by sender media or synthetic inducer. Therefore, the copy number of plasmids transfected into each individual cell varies greatly. As a result, the measured fluorescence intensity of individual cell would be partially determined by the copy number of transfected plasmids, instead of the actual induced activation.

To normalize for the difference in copy number, a constitutively expressed RFP was introduced into the receiver plasmid to serve as an internal control for the overall expression level of plasmid proteins that determined by plasmid copy number. In this case, cells transfected with more copies of receiver plasmid would have higher RFP intensity, and *vice versa*. Additionally, to control for the background YFP and RFP fluorescence that HEK293T cells may have, a sample with untransfected cells was included as well. Therefore, the output fluorescence of an individual cell (F_i) can be calculated by the following equation,

$$F_{\mathrm{i}} = \frac{F_{\mathrm{Y}} - F_{\mathrm{BY}}}{F_{\mathrm{R}} - F_{\mathrm{BR}}} \qquad (2)$$

where F_{Y} and F_{R} are the measured YFP and RFP fluorescence values, respectively, and F_{BY} and F_{BR} are the mean YFP and RFP fluorescence values of untransfected control sample, respectively. Finally, the output fluorescence of each sample can be calculated as the mean of the F_{i} values of all the cells within it.

Simple and complex cell-cell communication circuits

To ensure the activation performance and orthogonality of genetic circuit, we carefully selected the cell-cell communication systems. Several parameters were taken into consideration. (i) Each two systems must be orthogonal at the signal level, as shown in the signal orthogonality matrix. (ii) Each system must have acceptable performance, including basal output, maximal output and the resulting dynamic range. (iii) The sensitivity of receiver modules for different strains should be matched. If the sensitivity of one receiver module is considerably lower than other ones in the same circuit, it could slow down the response speed of the entire network.

Based on these criteria, we selected 5 orthogonal systems from our toolkit, including

the 3OC6, IV, pC, Sal and DAPG systems. All the 5 systems have acceptable dynamic range and exhibit no signal crosstalk among them. Although the DAPG and Sal systems have lower sensitivity comparing to the other three systems, they are selected because of their optimal orthogonality. Before constructing the two-channel communication circuit, the P_{tac} promoter which controls the expression of each regulator was replaced by a series of constitutive promoters, in order to adapt for the change in regulator expression level. The promoter which produces similar induction curve as the P_{tac} promoter was selected for constructing the circuit (Supplementary Figure 16).

Chemical synthesis of IV-HSL

In order to test the induction curve of IV receiver, we synthesized IV-HSL by coupling L-homoserine lactone hydrobromide with isovaleryl chloride under the condition of Et_3N at 0 °C to give isovaleryl-HSL in ~88% yield (Supplementary Figure 21a). Basically, L-homoserine lactone hydrobromide (50 mg) was dissolved in 3 mL CH_2Cl_2 (DCM) at 0 °C, Et_3N (70 mg) was then added. After stirring the mixture for 30 minutes, the isovaleryl chloride (40 mg) was added dropwise over 5 minutes. The reaction mixture was allowed to warm to room temperature, and the solution was stirred for 4 hours. It was then concentrated under reduced pressure, dissolved in ethyl acetate, and extracted sequentially with 1M sodium hydrogen carbonate solution, 1M potassium hydrogen sulfate sodium and saturated sodium chloride solution. After drying over anhydrous sodium sulfate, the solvent was removed *in vacuo*, and the crude product was purified by column chromatography on silica gel to yield isovaleryl-HSL (45 mg) (Supplementary Figure 21b).

Supplementary Discussion

In order to characterize the impact sender and receiver genes have on host cell, a comprehensive cell burden analysis was conducted by measuring the growth curves of each sender and receiver strain of cell-cell communication systems.

All the senders and receivers were diluted and cultured following the protocol as flow cytometric characterization experiments (Supplementary Figure 5) except the final culture step was extended from 12 to 24 hours to reveal the entire growth curve.

The senders' growth curves show that expression of sender genes leads to various extent of growth delay in host cells (Supplementary Figure 22). Comparing to the control sample, the Sal and DAPG sender genes show significant delay (10~12h) of entering log phase. Considering the known toxicity of DAPG towards bacteria, nematodes and fungi[18, 19], it is understandable that the growth of DAPG sender could be greatly slowed by its own product. Likewise, salicylate may slow the growth of bacteria by reducing the resistance to antibiotics[20]. However, the C4, 3OC6, C8, 3OC12, pC and MMF senders also show slight delay of growth (~3h) comparing to the control sample, although these signal molecules are not known to be toxic to bacteria cells. Thus, we believe such delay of growth can be attributed to the burden of expressing heterologous genes. Meanwhile, such heterologous gene expression might also have slowed the growth of DAPG and Sal senders. Interestingly, the IV and NG sender show no sign of growth delay, and they contain five and four sender genes, respectively, suggesting the number of genes have not yet become a problem to host cells at this point. Therefore, based on the growth curves of senders, we believe the growth of host cells can be affected by the burden of protein expression, the toxicity of product signal molecule, or the compound effect of both.

Comparing to senders, the receivers show much less burden to hosts. All the receiver cells enter log phase at similar time which is slightly later (~3h) comparing to the control sample, possibly due to the burden caused by the expression of aTFs.

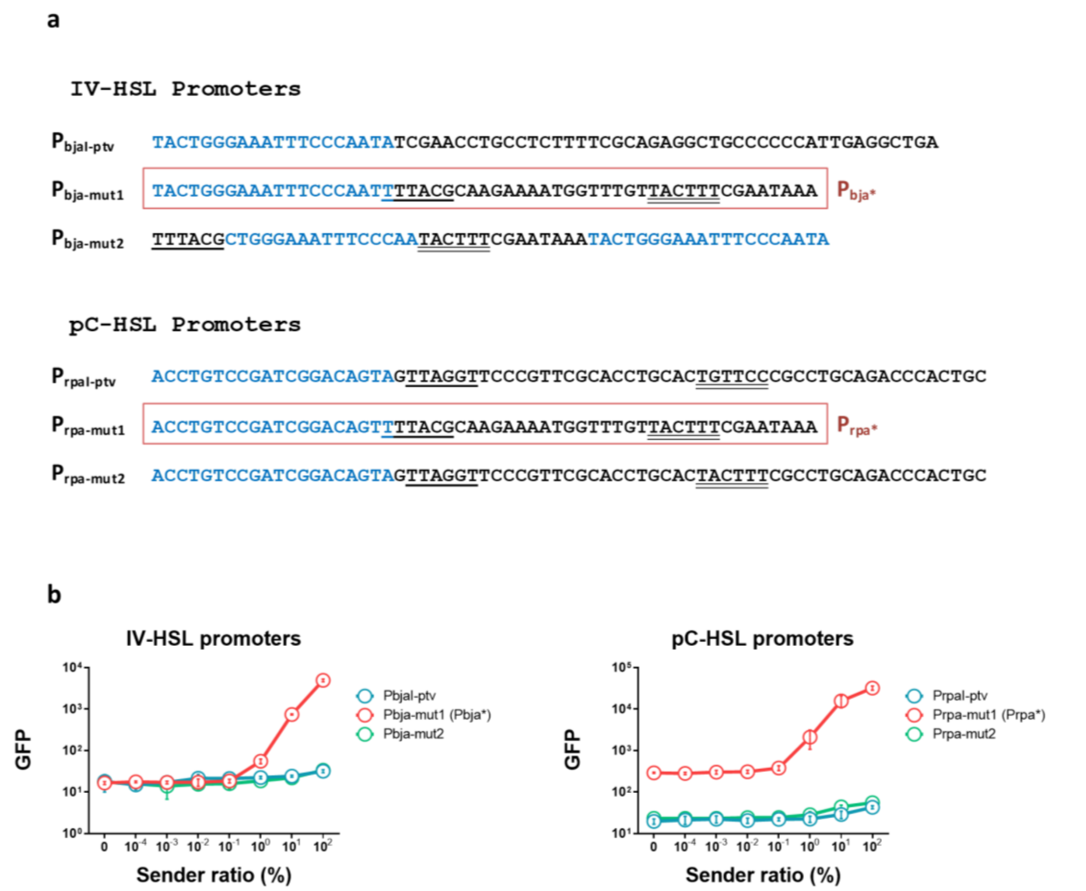

Supplementary Figure 1 Design and characterization of IV-HSL and pC-HSL responsive promoters. **a** Sequences of native and engineered promoters contain operators and a core *lux* promoter. Blue letters represent operators. Underlined and double underlined letters respectively indicate -35 and -10 region of each promoter, respectively. **b** sender-receiver cell cocultured response curves of the IV and pC systems. Data represent the mean fluorescence of three replicates, and error bar corresponds to the S.D. of each measurement. Source data are provided as a Source Data file.

a

No.	Name	Sequences
1	PmmfR1	TTGACAAACCTTCGGGAAGGTATGATACTCGAATAG
3	PmmfR3	TTGACAAACCTTCGGGAAGGTCTGATACTCGAATAG
4	PmmfR4	TTGACAAACCTTCGGGAAGGTGTGATACTCGAATAG
5	PmmfR5	TTGACATACCTTCGGGAAGGTATGATACTCGAATAG
6	PmmfR6	TTGACATACCTTCGGGAAGGTTTGATACTCGAATAG
9	PmmfR9	TTGACACACCTTCGGGAAGGTATGATACTCGAATAG
10	PmmfR10	TTGACACACCTTCGGGAAGGTTTGATACTCGAATAG
11	PmmfR11	TTGACACACCTTCGGGAAGGTCTGATACTCGAATAG
13	PmmfR13	TTGACAGACCTTCGGGAAGGTATGATACTCGAATAG
14	PmmfR14	TTGACAGACCTTCGGGAAGGTTTGATACTCGAATAG
15	PmmfR15	TTGACAGACCTTCGGGAAGGTCTGATACTCGAATAG
16	PmmfR16	TTGACAGACCTTCGGGAAGGTGTGATACTCGAATAG

b

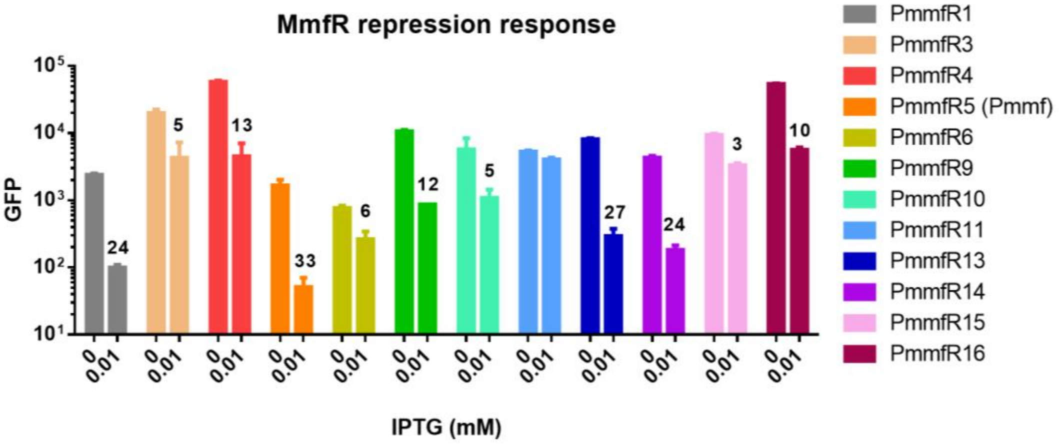

Supplementary Figure 2 The *de novo* design of responsive promoters for the MMF receiver. **a** Design of the P_{mmf} promoters. Blue letters indicate operator sequences. Letters of -35 and -10 regions are underlined. Highlighted letters indicate two varied nucleotides. **b** Each receiver cell was cultured with two different concentrations (0 and 0.01 mM) of IPTG before measured with flow cytometry. The data represent the mean fluorescence of three replicates, and error bar corresponds to the S.D. of each measurement. Source data are provided as a Source Data file.

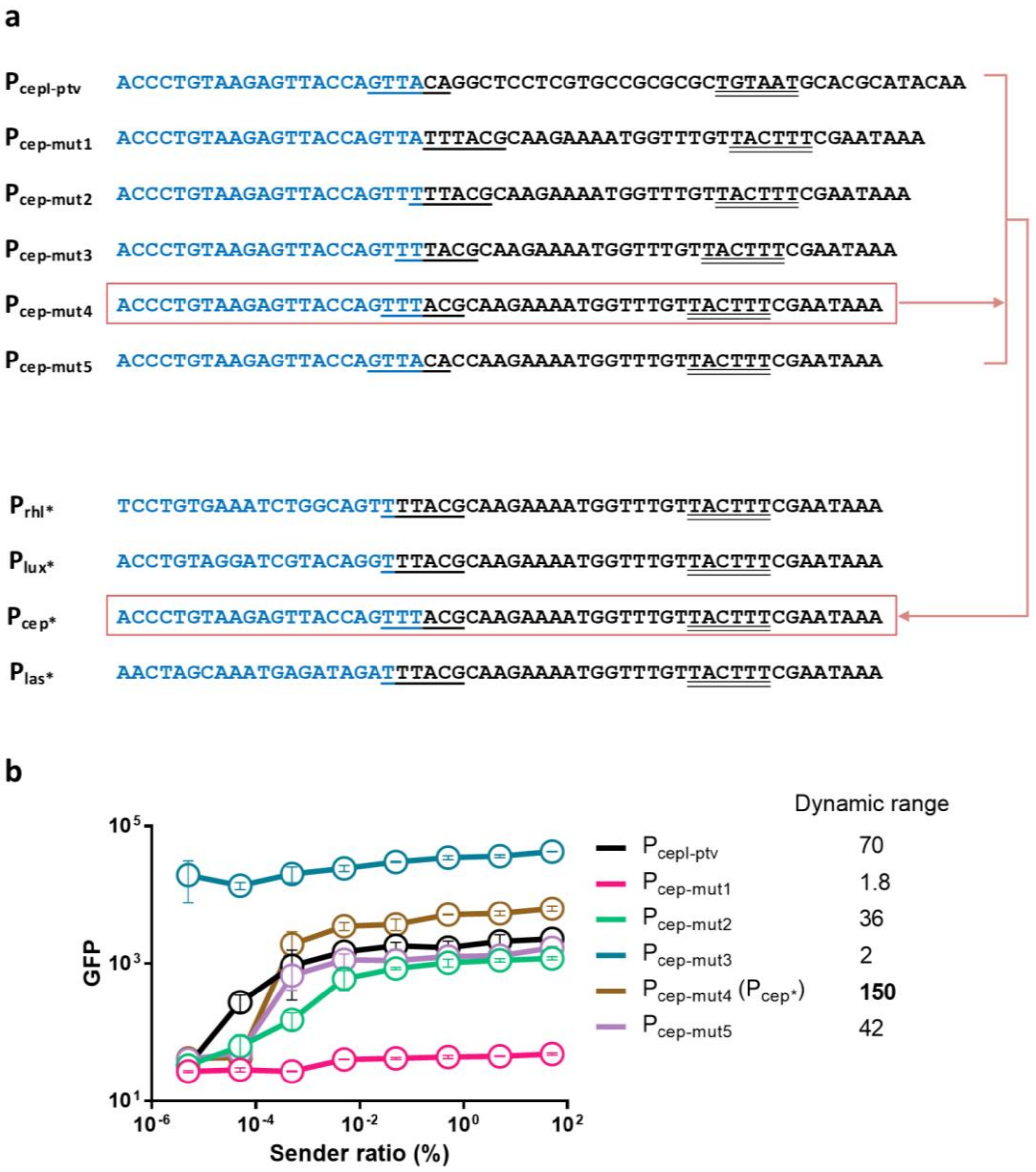

Supplementary Figure 3 Optimization of C8 cell-cell communication systems. **a** Sequences of native and engineered P_{cep} promoter (upper) and optimized promoter sequences for the four AHL based C4/3OC6/C8/3OC12 cell-cell communication systems (Lower). Blue letters represent the operator of each promoter. Underlined and double underlined letters indicate -35 and -10 region of each promoter, respectively. **b** Response curves and the corresponding dynamic ranges for the C8 system. Data represent the mean fluorescence of three replicates, and error bar corresponds to the S.D. of each measurement. Source data are provided as a Source Data file.

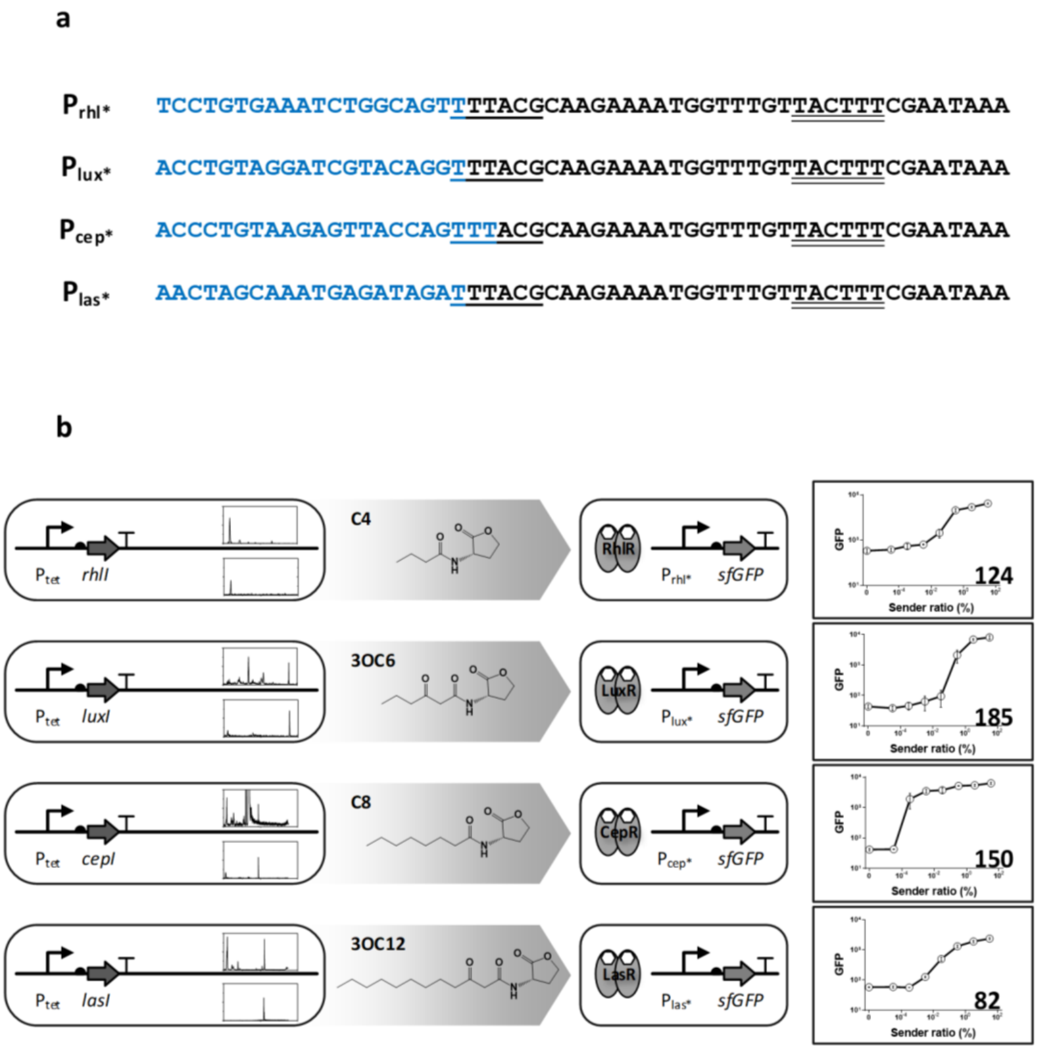

Supplementary Figure 4 Optimization and characterization of well-studied quorum sensing systems. **a** Optimized sequences for the four AHL based C4/3OC6/C8/3OC12 cell-cell communication systems. Blue letters represent the operator of each promoter. Underlined and double underlined letters indicate -35 and -10 region of each promoter, respectively. **b** The genetic circuit, structures of signaling molecules and induction curves of optimized systems. The HPLC-MS results of all signaling molecules were inserted in each sender figure. Data represent the mean fluorescence of three replicates, and error bar corresponds to the S.D. of each measurement. Source data are provided as a Source Data file.

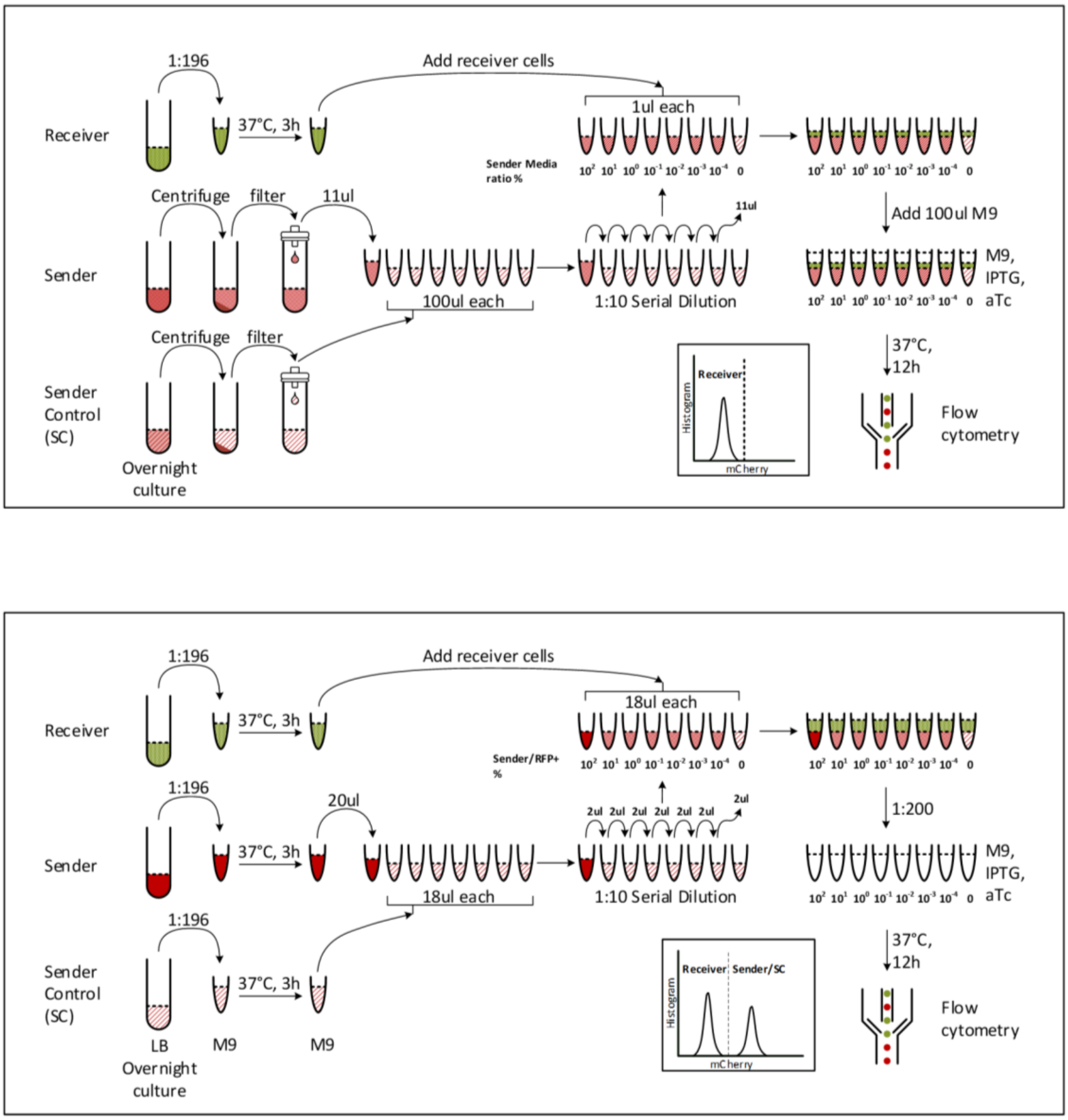

Supplementary Figure 5 Schematic representation of two different strategy for characterizing of cell-cell communication through sender media (Upper) or co-culture (Lower).

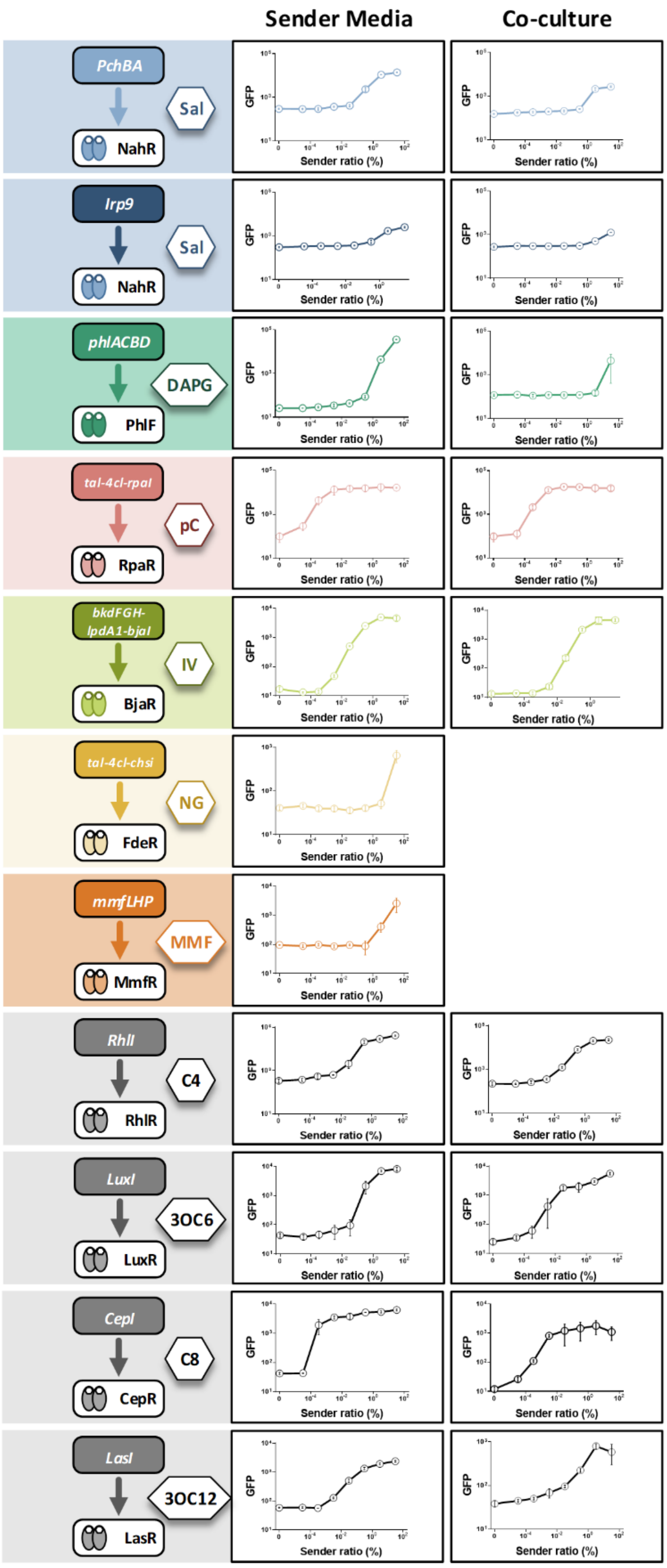

Supplementary Figure 6 Comparison of over-night cultured sender media and co-culture induction. The co-cultured results for the NG and MMF systems were not obtained. Data represent the mean fluorescence of three replicates, and error bar corresponds to the S.D. of each measurement. Source data are provided as a Source Data file.

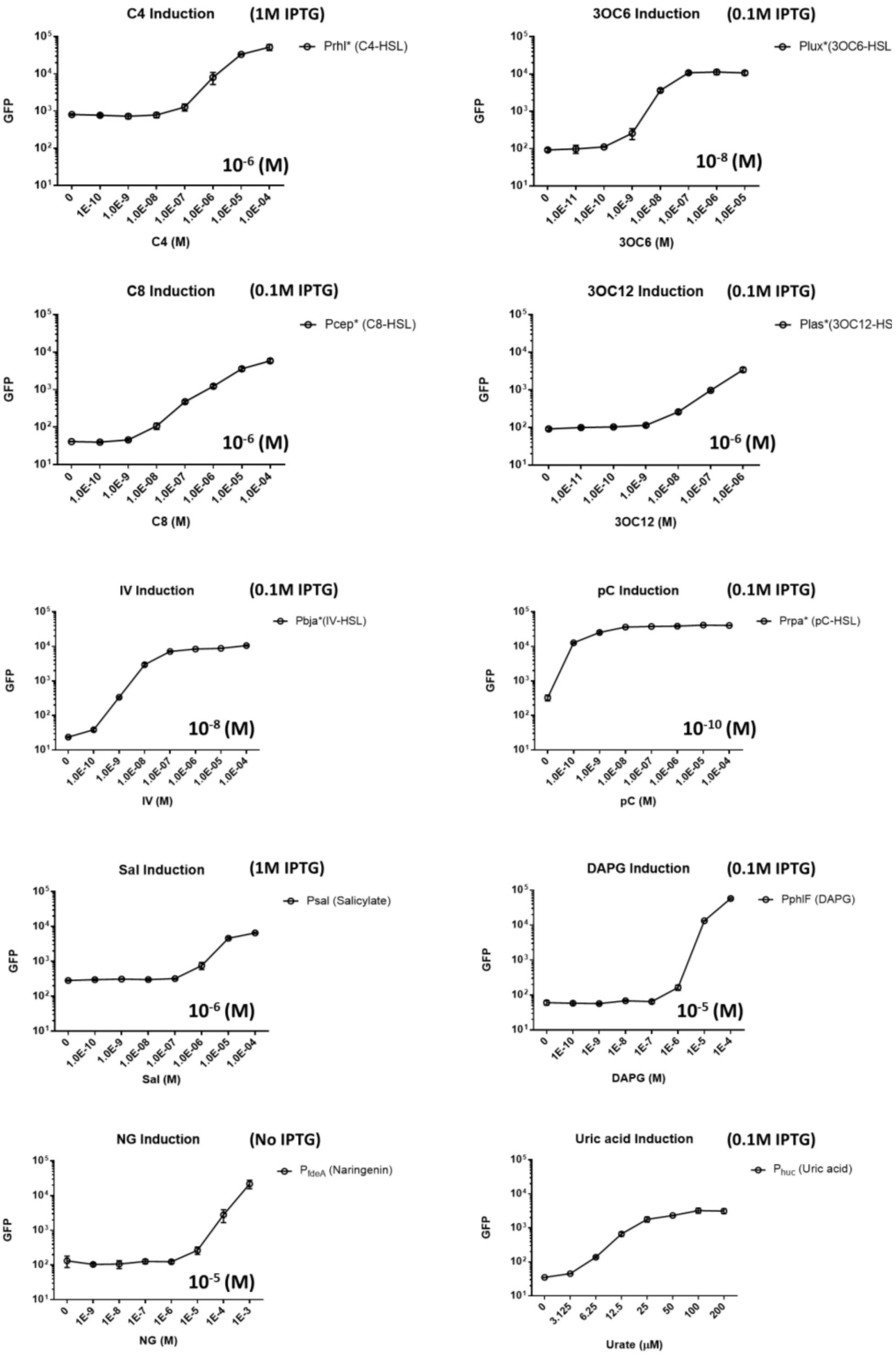

Supplementary Figure 7 Dose-response curves of receiver cells by chemically synthetized signal molecules. The EC50 value of each curve is shown. Data represent the mean fluorescence of three replicates, and error bar corresponds to the S.D. of each measurement. Source data are provided as a Source Data file.

a

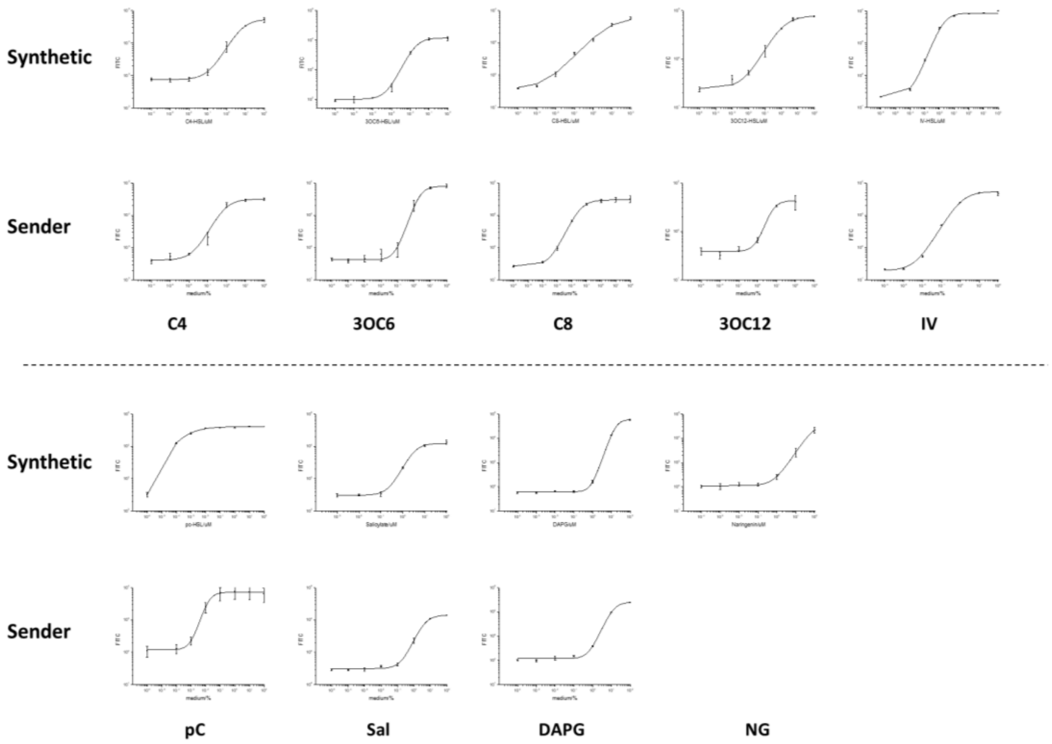

b

Synthetic (uM)	C4	3OC6	C8	3OC12	IV	pC	Sal	DAPG	NG
**EC10**	0.8532	0.0053	0.5866	0.0848	0.0013	0.0000	1.3025	6.4964	19.3337
**EC50**	6.7665	0.0195	6.3315	1.4368	0.0301	0.0004	6.0125	17.4050	51.0516
**EC90**	54.2496	0.1293	122.2931	24.4633	0.7547	0.0149	29.5482	46.7686	110.8882

Sender (%) (x)	C4	3OC6	C8	3OC12	IV	pC	Sal	DAPG
**EC10**	0.0467	0.5369	0.0040	1.2296	0.1288	0.0036	0.7081	3.7014
**EC50**	0.7407	3.9714	0.0502	6.1635	0.9712	0.0191	3.8558	14.9508
**EC90**	1.8623	9.7599	0.3246	18.7259	10.1870	0.0675	13.0357	46.0406

Supplementary Figure 8 Fitting of the activation curves of each receiver cells induced by chemically and biologically synthesized signal molecules. **a** The induction curves for chemically synthetic signaling molecules or co-cultured sender-receiver systems. **b** The EC10, EC50, EC90 values were calculated based on the above curves.

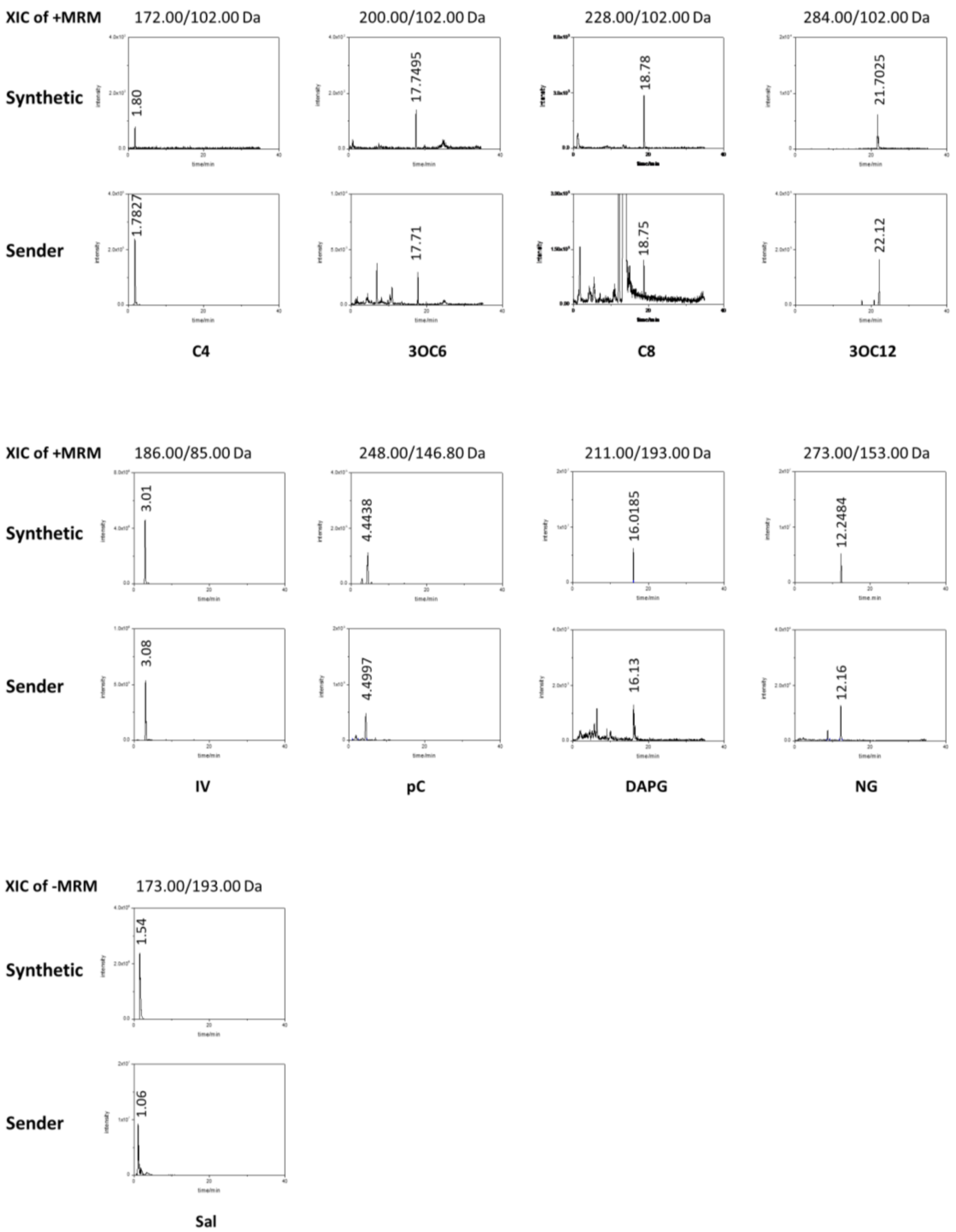

Supplementary Figure 9 Quantitation of the yield of each signaling molecule by HPLC-MS. Each signaling molecule from the cell-free culture medium of sender cell was quantitatively characterized by HPLC-MS with MRM (multiple reaction monitoring), using the cognate synthetic chemical as control. Number above each peak indicates time (min) of the peak. Numbers above each graph indicate the MRM ion pairs.

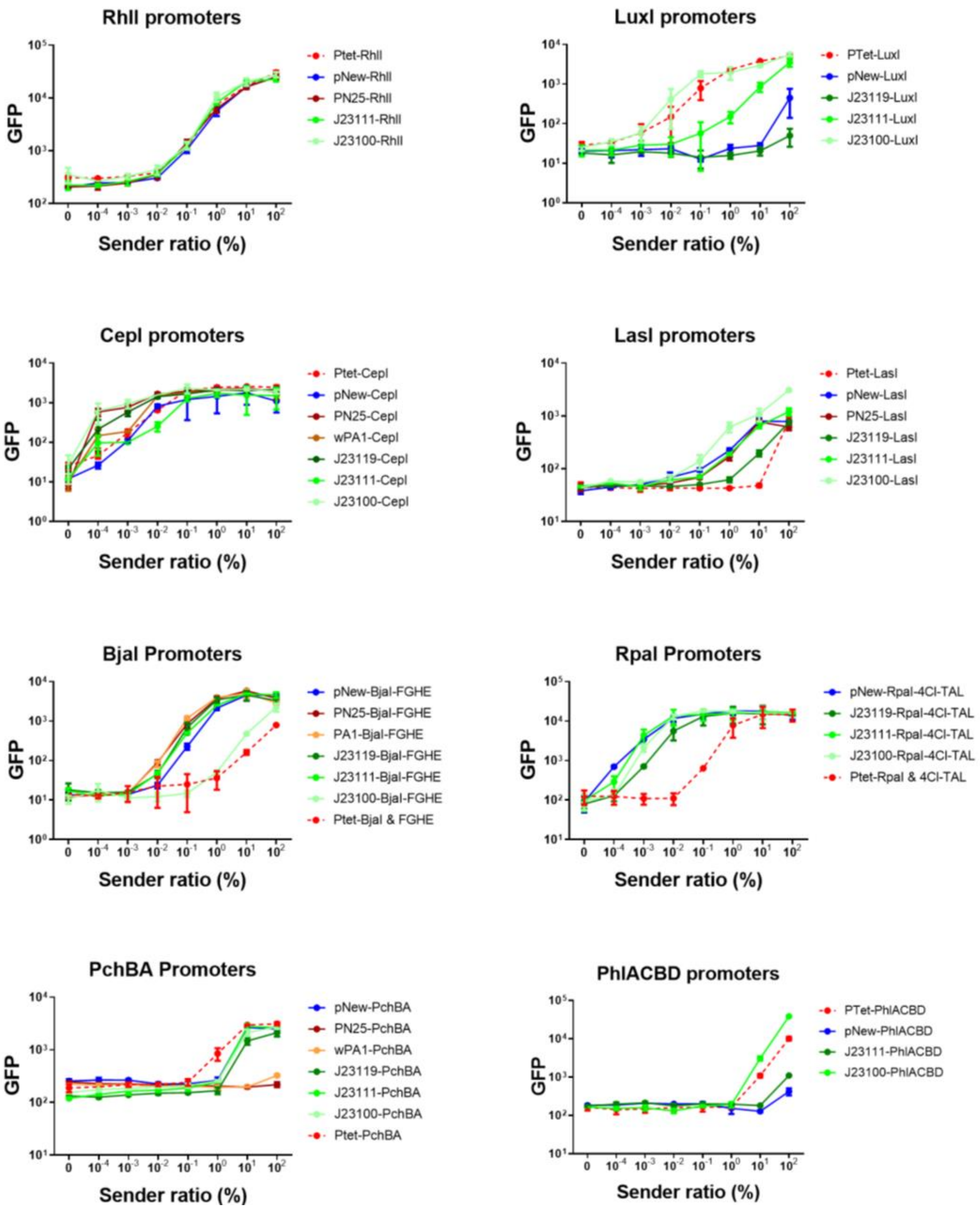

Supplementary Figure 10 Optimization of the biosynthetic capacity of senders. Sender with each promoter was diluted to the indicated percentages and co-cultured with cognate receiver. Fluorescence of each sample was measured by flow cytometry. A same constitutive and inducible promoter are used to vary the expression of the biosynthesis gene cassettes. Abbreviations: (PA1, PN25: Virus originated promoters; pNew: P_{tet}-type inducible promoter with CymR as the repressor; FGHE: the bkdFGH-IpdA gene cluster) The data represent the mean fluorescence of three replicates, and error bar corresponds to the S.D. of each measurement. Source data are provided as a Source Data file.

a

CMV1 Promoter sequence

GGTACCACCTGTCCGATCGGACAGTTGATATCACCTGTCCGATCGGACAGTTCTCGAGAGCTCGGTACCCGGGTCGA

RpaR binding site **RpaR binding site**

GGTAGGCGTGTACGGTGGGAGGCCTATATAAGCAGAGCTCGTTTAGTGAACCGTCAGATCGCCTGGAGACGCCATCC

Minimal CMV promoter **Inr**

ACGCTGTTTTGACCTCCATAGAAGACACCGGGACCGATCCAGCCTCCGCGGC

CMV3G Promoter sequence

GGTACCACCTGTCCGATCGGACAGTTGATATCACCTGTCCGATCGGACAGTTCTCGAGAGCCTCGGTACCCGGGTCG

RpaR binding site **RpaR binding site**

AGGTAGGCGTGTACGGTGGGCGCCTATAAAAGCAGAGCTCGTTTAGTGAACCGTCAGATCGCCTGGAGCAATTCCAC

Minimal CMV promoter

AACACTTTTGTCTTATACCAACTTTCCGTACCACTTCCTACCCTCGTAAA

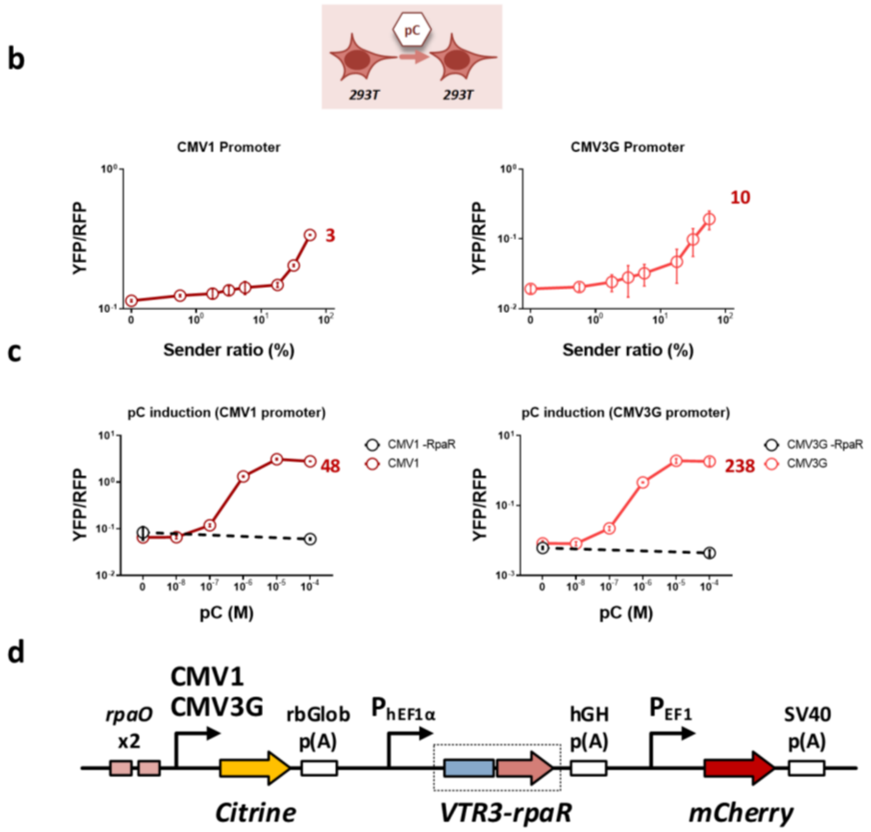

Supplementary Figure 11 Intercellular communication in mammalian cells. **a** The sequence of engineered responsive promoters for mammalian cells. **b** Response curves of CMV1 (left) or CMV3G (right) promoters in the 293T cell line by the cultured 293T sender cell medium. **c** Induction curves of 293T receivers with CMV1 (left) or CMV3G (right) promoters by chemical synthesized pC-HSL. Data represent the mean fluorescence of three replicates, and error bar corresponds to the S.D. of each measurement. **d** Diagram of the mammalian receiver plasmids. Source data are provided as a Source Data file.

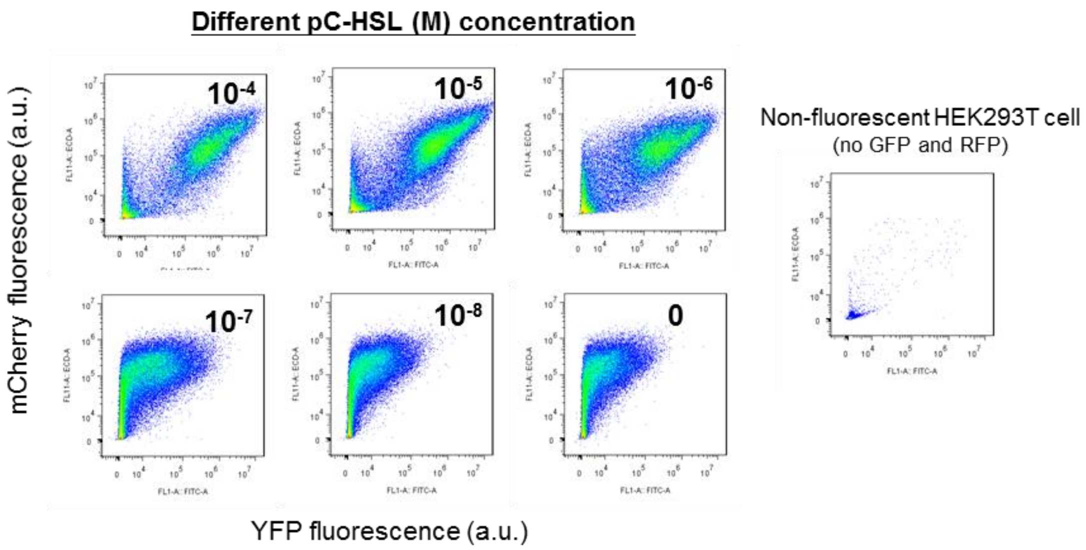

Supplementary Figure 12 FACS data of mammalian pC receiver activation. Non-fluorescent HEK-293T cell was the negative control.

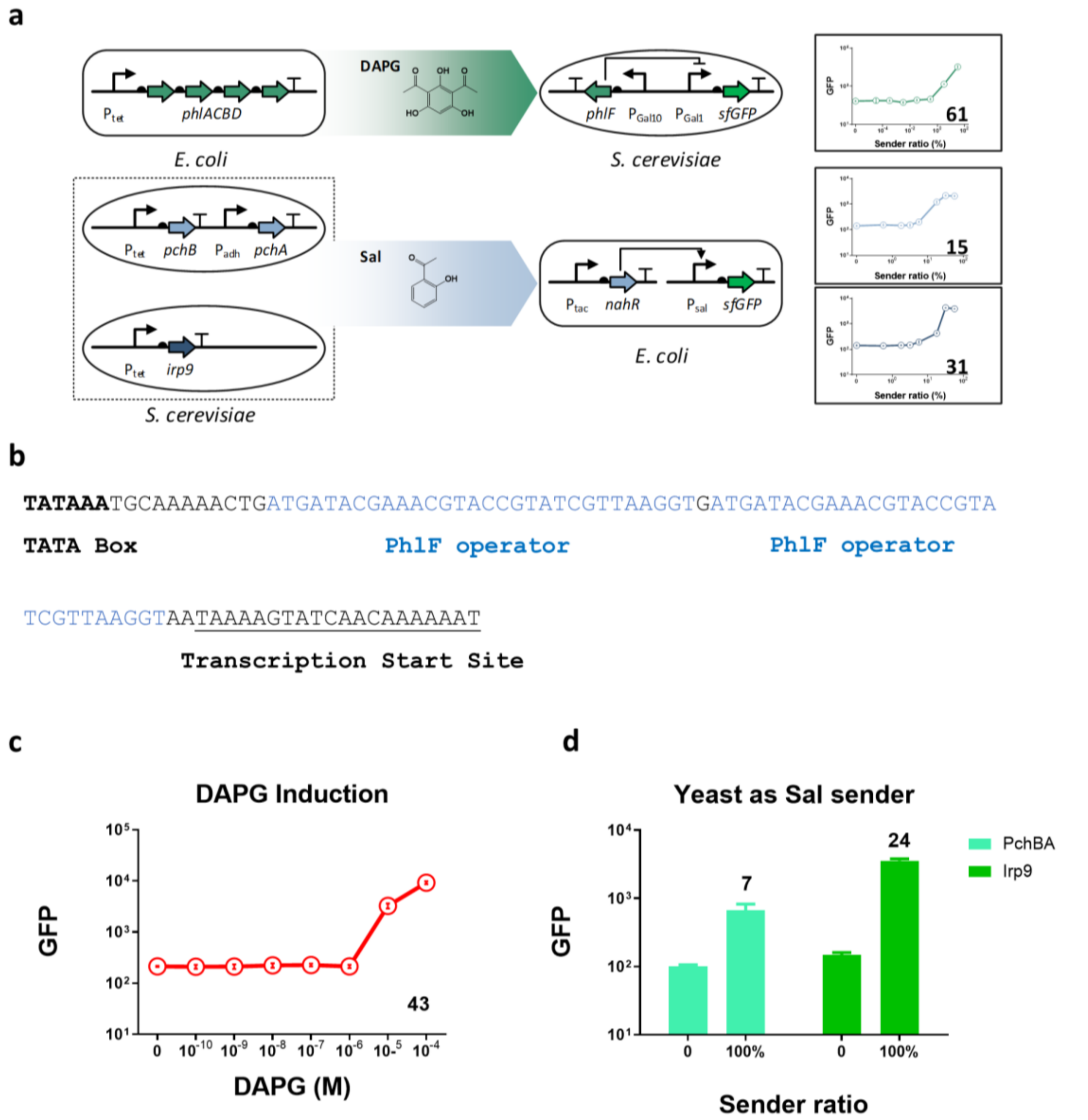

Supplementary Figure 13 Cross-kingdom communication between E. coli and S. cerevisiae. **a** The genetic circuit, structures of signaling molecules and induction curves of receiver cells in the cross-kingdom communication between S. cerevisiae and E. coli. **b** sequence of $P_{PhlF\text{-}Y}$ promoter. **c** Response curves of S. cerevisiae receiver by synthetic DAPG. **d** Fluorescent response of E. coli Sal receiver with the cell-free culture medium of S. cerevisiae Sal sender with PchBA or Irp9. Data represent the mean fluorescence of three replicates, and error bar corresponds to the S.D. of each measurement. Source data are provided as a Source Data file.

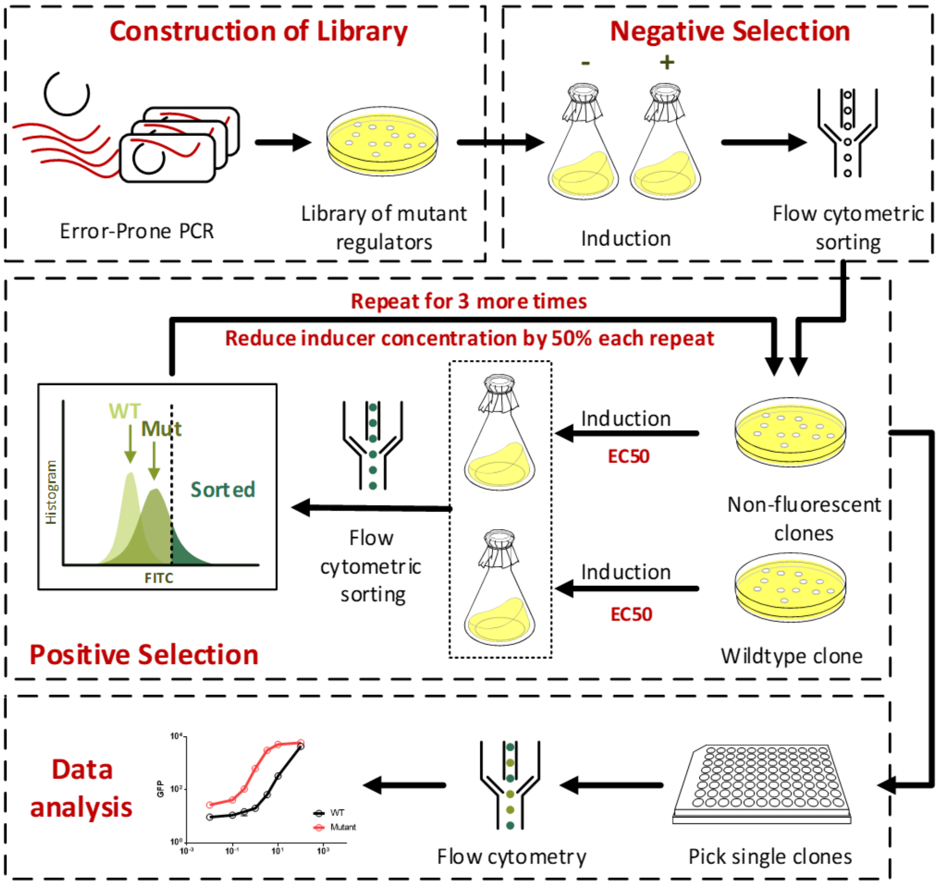

351

352 **Supplementary Figure 14** Optimization of the sensitivity of DAPG and salicylate
353 systems by directed evolving PhlF and NahR protein.

a

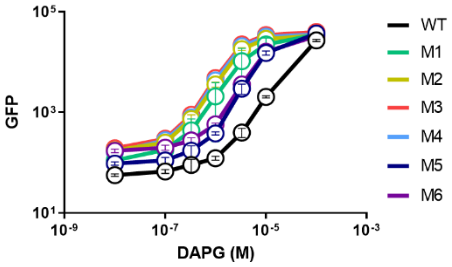

	EC50 Fold change	Mutations	Dynamic Range
WT	-	-	440
M1	13.810	**D128G**	210
M2	13.951	**D128G**, Q181R	222
M3	13.609	**D128G**, T24A	267
M4	14.127	**D128G**, D154G	188
M5	3.784	K127N, V194A	353
M6	4.024	E132D	209

b

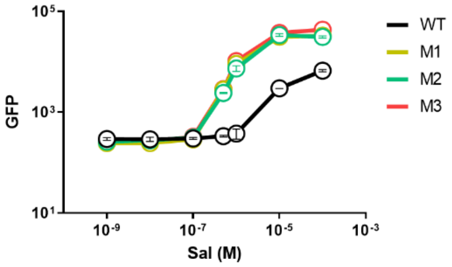

	EC50 Fold change	Mutations	Dynamic Range
WT	-	-	26.5
M1	15.925	H57Q; **Q168R**; V216A	137.1
M2	14.250	H57Q; **Q168R**; V267D	109.9
M3	15.175	I25V; **Q168R**	139.6

Supplementary Figure 15 Optimized PhlF and NahR mutants with increased sensitivity to the DAPG and Sal molecules, respectively. **a** The induction curves and mutated amino acids of each PhlF mutant. **b** The induction curves and mutated amino acids of each NahR mutant. Data represent the mean fluorescence of at least three replicates, and error bars show the S.D. of each measurement. Source data are provided as a Source Data file.

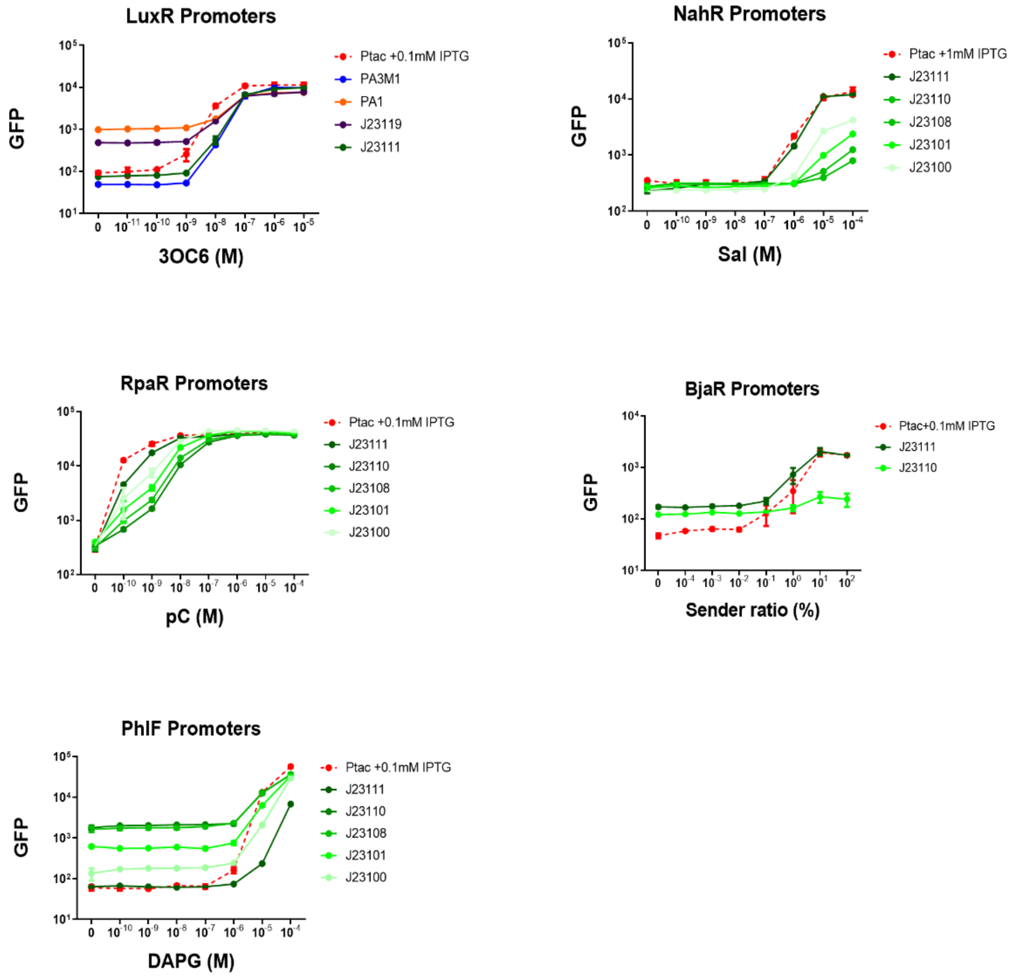

Supplementary Figure 16 Adaptation of promoters for expressing regulators of each receiver of intercellular communication circuit. Data represent the mean fluorescence of three replicates, and error bars show the S.D. of each measurement. Source data are provided as a Source Data file.

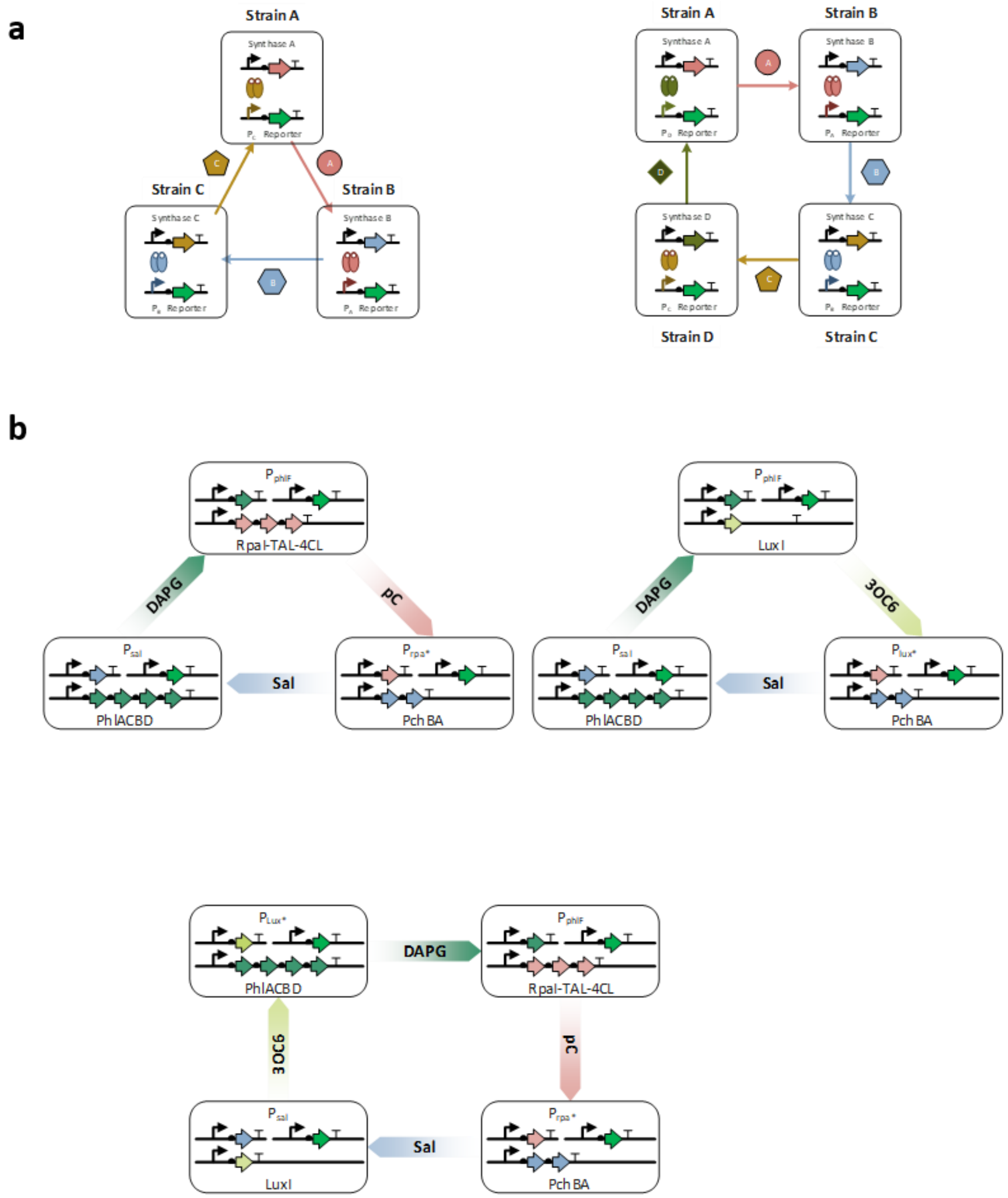

Supplementary Figure 17 Diagrams of intercellular signal transduction with three and four cell-cell communication systems. **a** Diagram of the three- and four-channel communication loops. **b** The genetic constructs of workable three- and four-channel communication loops.

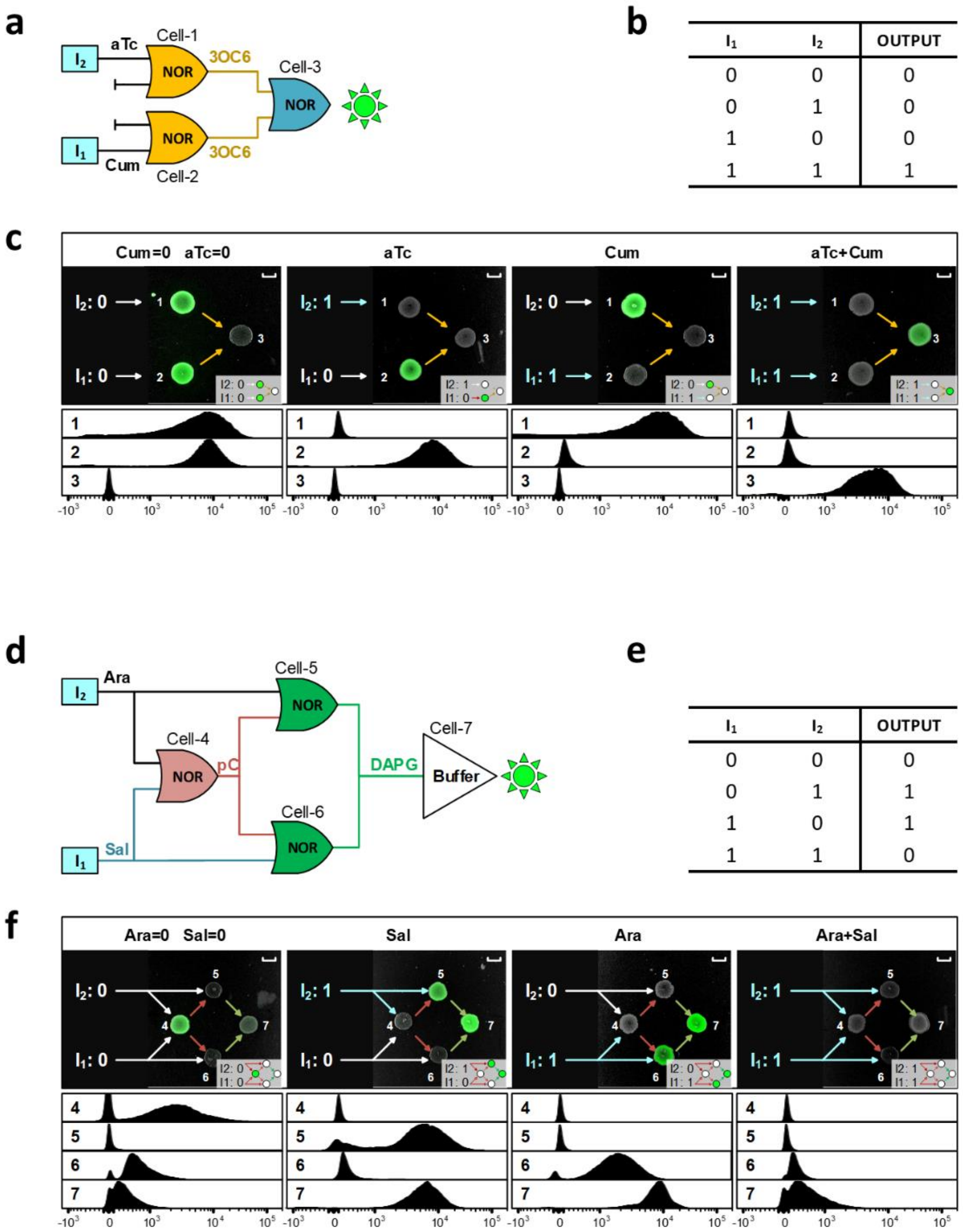

Supplementary Figure 18 Bio-computing circuits with the functions as AND or XOR logic gates. **a and d** Genetic circuit of AND (a) and XOR (d) gates. **b and e** Truth table of AND (b) and XOR (e) gates. **c and f** The distributed bio-computing results for the AND (c) and XOR (f) gates. The scale bars correspond to 0.5cm.

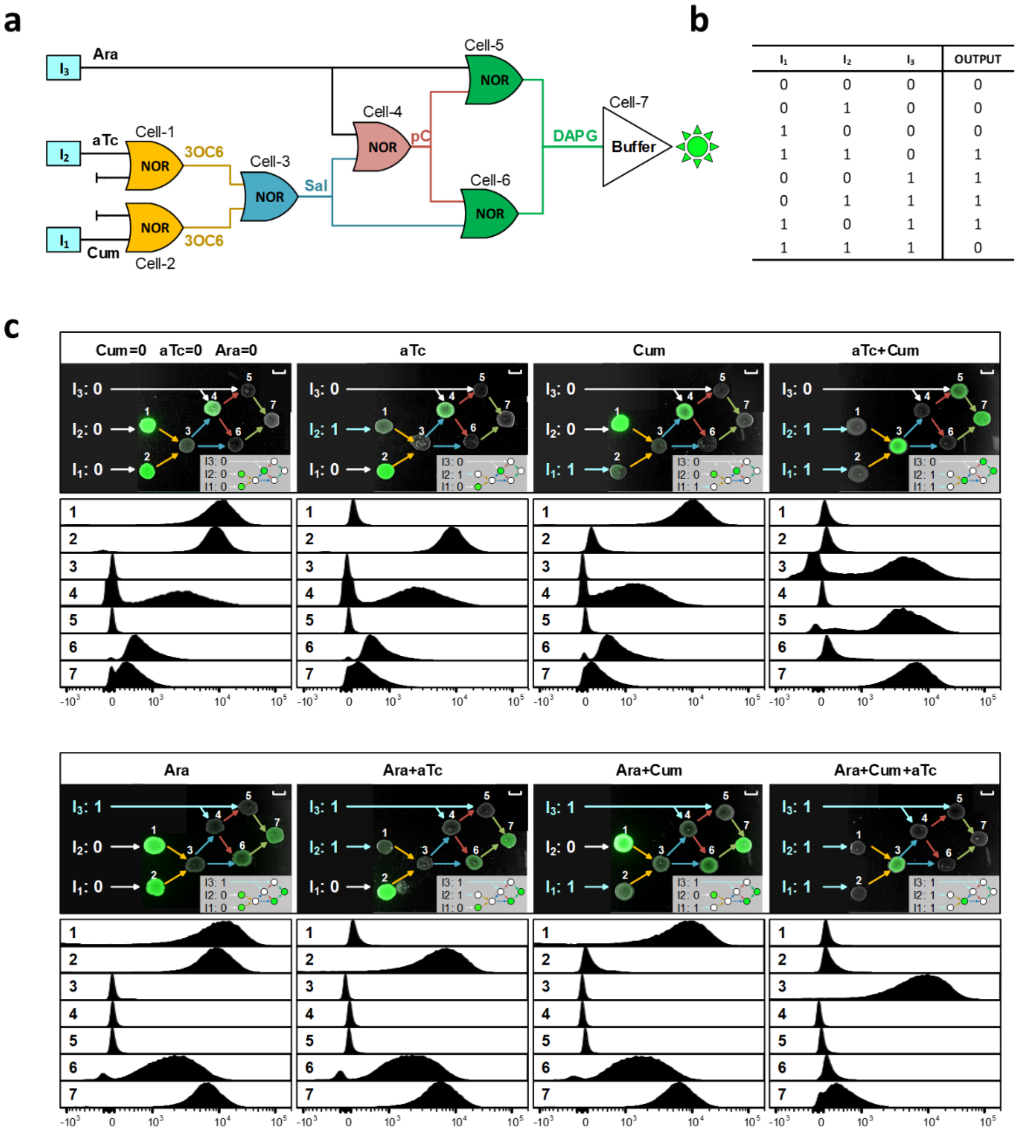

I_1	I_2	I_3	OUTPUT
0	0	0	0
0	1	0	0
1	0	0	0
1	1	0	1
0	0	1	1
0	1	1	1
1	0	1	1
1	1	1	0

Supplementary Figure 19 Design and construction of genetic bio-computing circuits with a complex 3-input AND-XOR logic gate function. **a and b** Genetic circuit (a) and truth table (b) of 3-input AND-XOR logic gates. **c** The distributed bio-computing results for the 3-input AND-XOR logic gates. The scale bars correspond to 0.5cm.

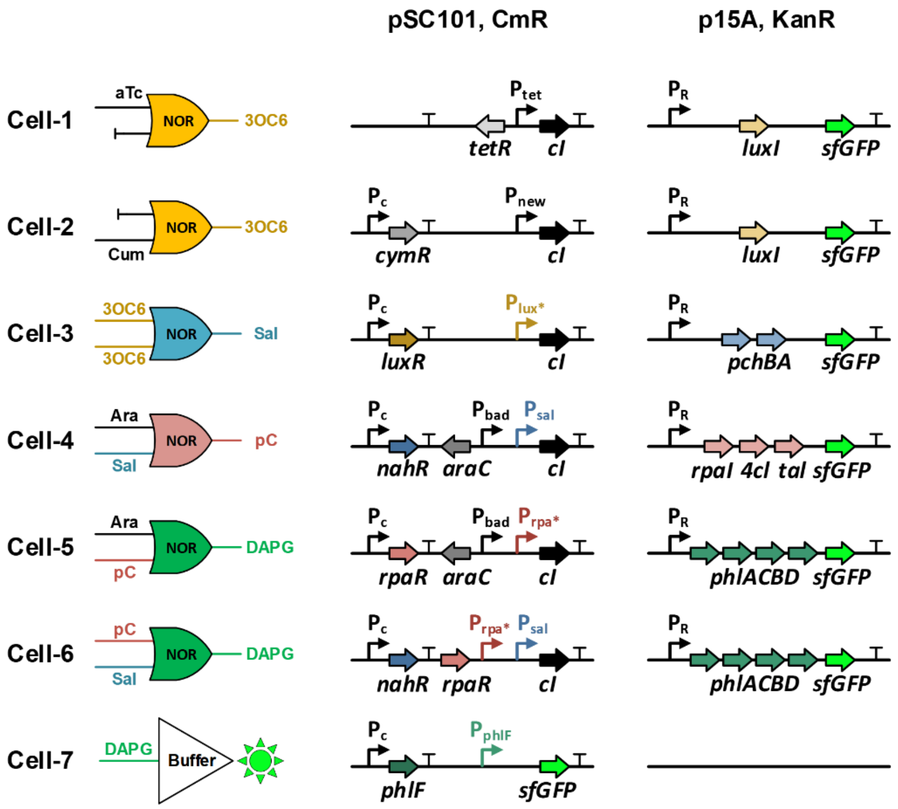

Supplementary Figure 20 Diagram of the genetic circuit of NOR gates in AND-XOR logic gates.

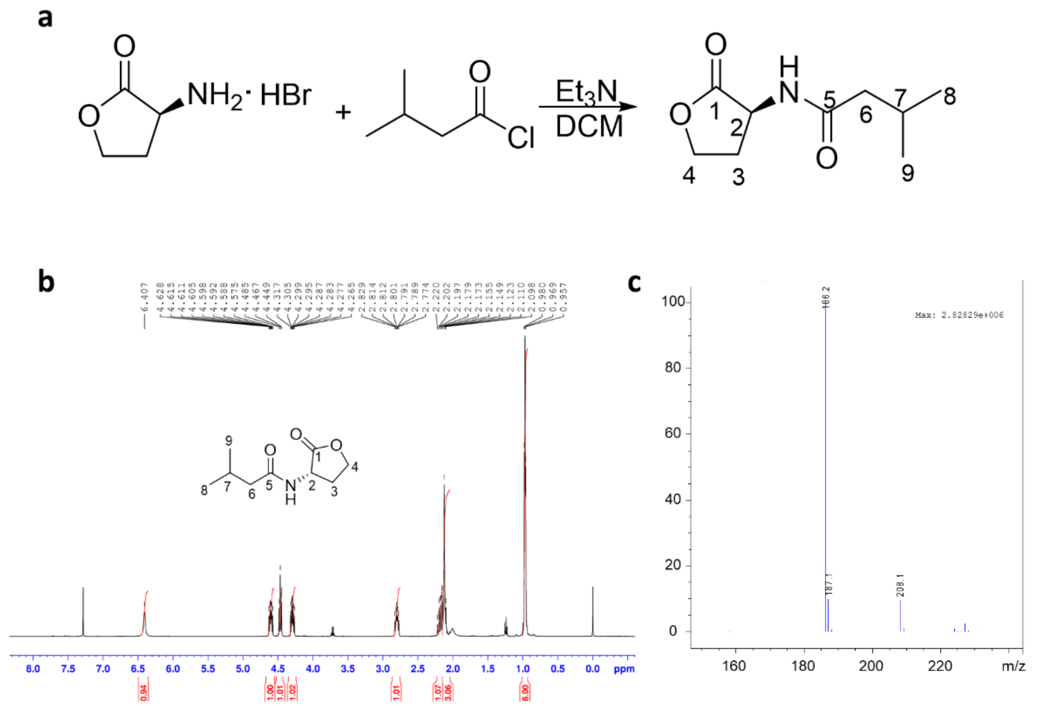

^{1}H NMR (500 MHz, CDCl3): δ = 0.97 (d, J = 5.35 Hz, 6H, H-8, 9), 2.10-2.20 (m, 4H), 2.80 (m, 1H), 4.47 (t, J = 8.7Hz, 1H, H-2), 4.60, 4.29 (m, 2H, H-4). MS (ESI): m/z = 186.2 [M + H] $^{+}$.

Supplementary Figure 21 Chemical synthesis of IV-HSL. **a** The reaction equation and description of the synthesis of IV-HSL. **b and c** Spectroscopic analysis of the synthesized IV-HSL through NMR (b) and mass-spectrometry (c).

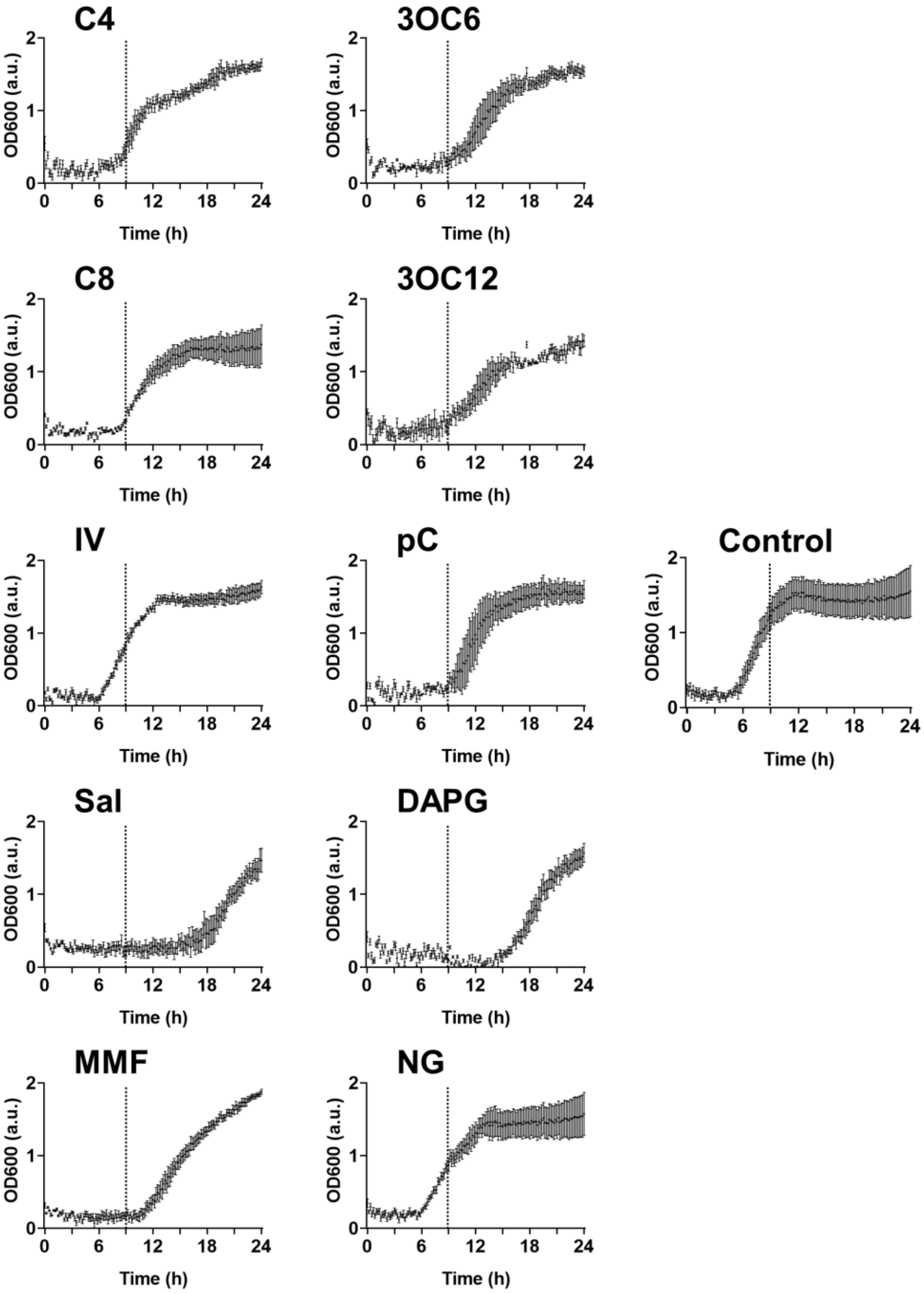

Supplementary Figure 22 Growth curve of each sender. Control sample were transformed with empty vectors with the same antibiotic resistance and replicons. Dashed line indicates 9 hours. Data represent the mean fluorescence of at least three replicates, and error bars show the S.D. of each measurement. Source data are provided as a Source Data file.

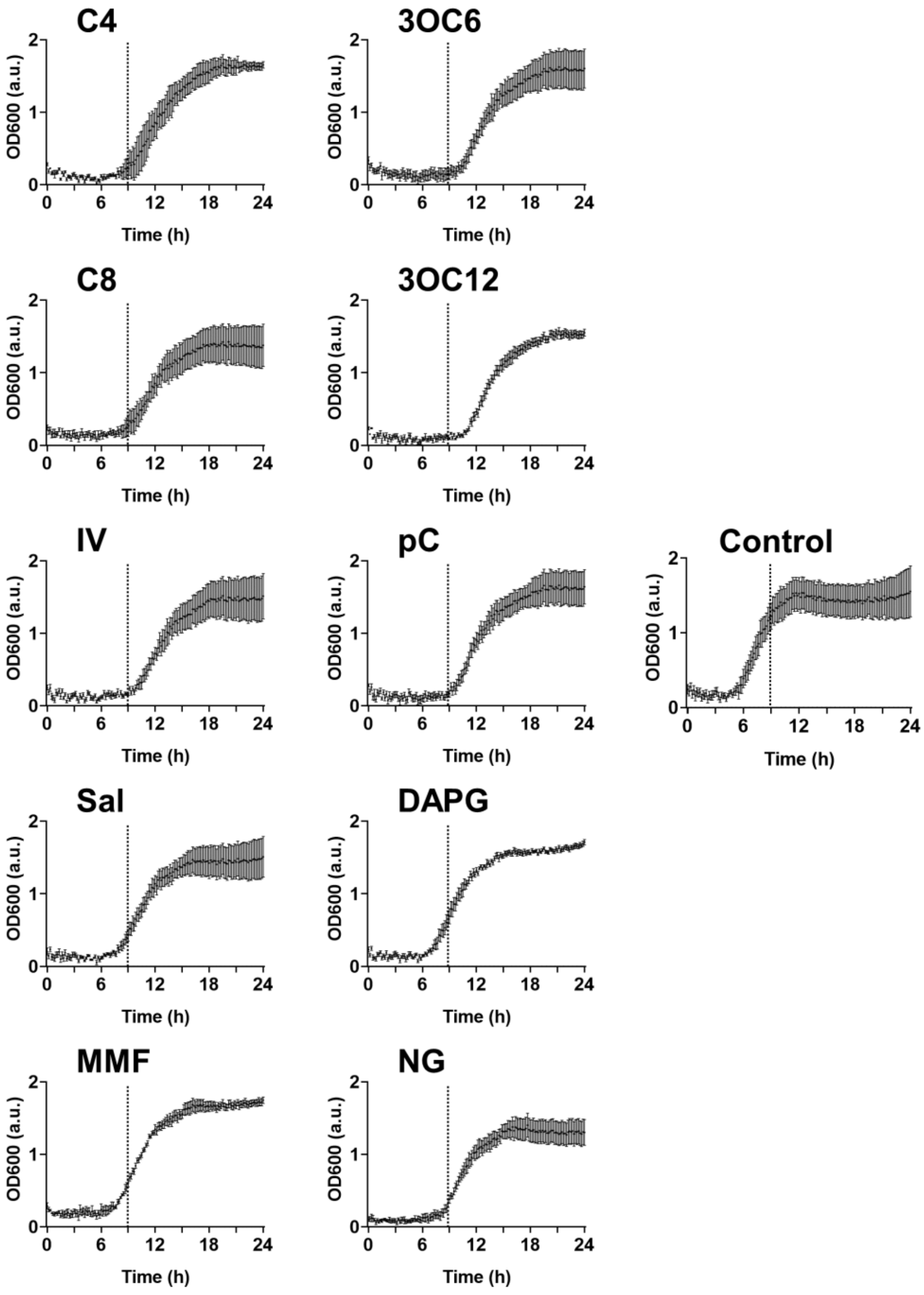

Supplementary Figure 23 Growth curve of each receiver. Control sample were transformed with empty vectors with the same antibiotic resistance and replicons. Dashed line indicates 9 hours. Data represent the mean fluorescence of at least three replicates, and error bars show the S.D. of each measurement. Source data are provided as a Source Data file.

Inducer	Synthesis genes	EC (KEGG)	Receptor genes	Promoter	Source
**DAPG**	*phlD*<br>*phlACB*	2.3.1.253<br>2.3.1.272	*phlF*	P_{phlF}	*P. fluorescens*
**pC**	*tal* (1)<br>*4cl* (2)<br>*rpaI* (2)	4.3.1.23<br>6.2.1.12<br>2.3.1.229	*rpaR* (2)	P_{rpa*}	(1) *R. Sphaeroides*<br>(2) *R. Palustris*
**IV**	*bdkFGH* (1)<br>*lpdA1* (1)<br>*bjaI* (2)	1.2.4.4<br>1.8.1.4<br>2.3.1.228	*bjaR* (2)	P_{bja*}	(1) *S. avermitilis*<br>(2) *B. Japonicum*
**Sal**	*pchB* (2)<br>*pchA* (2)<br>*irp9* (3)	4.2.99.21<br>5.4.4.2<br>4.2.99.21	*nahR* (1)	P_{sal}	(1) *P. putida*<br>(2) *P. aeruginosa*<br>(3) *Y. enterocolitica*
**MMF**	*mmfLHP*	/	*mmfR*	P_{mmf}	*S. coelicolor*
**NG**	*tal* (1)<br>*4cl* (2)<br>*chs* (3)<br>*chi* (4)	4.3.1.23<br>6.2.1.12<br>2.3.1.74<br>5.5.1.6	*fdeR* (5)	P_{fdeA}	(1) *R. Sphaeroides*<br>(2) *R. Palustris*<br>(3) *Petunia X hybrid*<br>(4) *Medicago sativa*<br>(5) *H. Seropedicae*
**Uric acid**	*xdhABC* (1)	1.17.1.4	*hucR* (2)<br>*ygfU* (3)	P_{hucR}	(1) *R. capsulatus*<br>(2) *D. Radiodurans*<br>(3) *E. coli*
**Pyrone**	*bkdA*<br>*bkdBC*<br>*ppyS*<br>*ngrA*	1.2.4.4<br>2.3.1.168<br>2.3.1.153<br>2.7.8.7	*pluR*	P_{pcfA}	*P. luminescens*
**A-factor**	*afsA*	/	*arpA*	P_{arp}	*S. griseus*
**SCB1**	*scbA*	/	*scbR*	P_{scb}	*S. coelicolor*
**C4**	*rhlI*	2.3.1.184	*rhlR*	P_{rhl*}	*P. aeruginosa*
**3OC6**	*luxI*	2.3.1.184	*luxR*	P_{lux*}	*V. fischeri*
**C8**	*cepI*	2.3.1.184	*cepR*	P_{cep*}	*B. cenocepacia*
**3OC12**	*lasI*	2.3.1.184	*lasR*	P_{las*}	*P. aeruginosa*

Supplementary Table 1 Information of *de novo* designed and optimized cell-cell communication systems. Numbers of index indicate the organism which the gene(s) originates.

Name	Promoter sequence
P_{phlF}	GATTCGTTACCAA**TTGACA**TGATACGAAACGTACCG**TATCGT**TAAGGTTACTAGAG
P_{rpa*}	ACCTGTCCGATCGGACAGT**TTTACG**CAAGAAAATGGTTTGT**TACTTT**CGAATAAA
P_{bja*}	TACTGGGAAATTTCCCAAT**TTTACG**CAAGAAAATGGTTTGT**TACTTT**CGAATAAA
P_{sal}	GGGGCCTCGCTTGGGTTATTGCTGGTGCCCGGCCGGGCGCAATATTCATGTTGAT<br>GATTTATTATATATCGAGTGGTGTAT**TTATCA**ATATTGTTTGCTCCGT**TATCGT**T<br>ATTAACAAGTCATCAATAAAGCCATCACGAGTACCATAGAGGATC
P_{mmf}	**TTGACA**TACCTTCGGGAAGGTAT**GATACT**CGAATAG
P_{fdeA}	CCTGTTGTGCTTGTTCTTGCCGACCCTCGGATAGACGACGGATGGGGTGGTCAAT<br>GTATTGATGCCGTCCATATCATGAATCAAAACAATCCAT**TTGATC**AATATCAAGC<br>TCACTCT**TAAGCT**TCACTCATCCGCTGCATGGCCCCACCAGAAAGGGCTGGCGCG<br>GCAAGCCGGCGGCGCACTCGCACTGGATGCGCCGCTGTTGAGCCTGGCCATGACA<br>ACGCGCCGATAGCGGCCACACCCCGCCAGGCAGGGTAGGAGACAAGGAG
P_{hucR}	**TTTACA**TAGGTAGACATCTAAGTA**TATGTT**GTGTGGAACCGTCACACAG
P_{rhl*}	TCCTGTGAAATCTGGCAGT**TTTACG**CAAGAAAATGGTTTGT**TACTTT**CGAATAAA
P_{lux*}	ACCTGTAGGATCGTACAGG**TTTACG**CAAGAAAATGGTTTGT**TACTTT**CGAATAAA
P_{cep*}	ACCCTGTAAGAGTTACCAG**TTTACG**CAAGAAAATGGTTTGT**TACTTT**CGAATAAA
P_{las*}	AACTAGCAAATGAGATAGA**TTTACG**CAAGAAAATGGTTTGT**TACTTT**CGAATAAA

Supplementary Table 2 Sequences of engineered responsive promoters for each cell-cell communication system, other native responsive promoters are not shown.

Parameter	Negative Mode	Positive Mode
Ion spray (IS) potential	4.5 kV	5.5 kV
Nebulizer Gas (GS1)	50 psi	60 psi
bath gas (GS2)	50 psi	60 psi
Curtain gas (CUR)	20 psi	20 psi
Source temperature (TEM)	500 °C	500 °C
Declustering potential (DP)	-110 V	80 V
Entrance potential (EP)	-10 eV	10 eV
Collision energy (CE)	-20 eV	40 eV (DAPG 20 eV)
Collision exit cell potential (cxp)	-18 eV	13 eV
Dwell time	200 msec	200 msec

Supplementary Table 3 Parameters for HPLC-MS/MRM in quantification of yield of signaling molecules.

Primer Name	Primer Sequence
RhlI_FW1	CAGGTCTCCATGATCGAACTGCTGTCCGA
RhlI_RV1	CAGGTCTCCTCAAACAGCCATGGACAGCG
LuxI_FW1	CAGGTCTCCATGACTATAATGATAAAAAAATCGGATTTTTTG
LuxI_RV1	CAGGTCTCCTCAATTTAAGACTGCTTTTTTAAACTGTTCATT
CepI_FW1	CAGGTCTCCATGCAGACCTTTGTGCATGAA
CepI_RV1	CAGGTCTCCTTAAGCTGCGATTGCTTGACGG
LasI_FW1	CAGGTCTCCATGATCGTTCAGATCGGTCG
LasI_RV1	CAGGTCTCCTTAGGAAACAGCCAGACGCT
BjaI_FW1	CAGGTCTCCATGGGCGTGAGTATGATTCATG
BjaI_RV1	CAGGTCTCCTTAGGCAGATTTGCGTTGCG
bkdFGHE_FW1	ACCCGTTTTTTGGGCTAACAGG
bkdFGHE_RV1	TTAGTCGTGAGAATGCAGCGGCTT
RpaI_FW1	CAGGTCTCCATGCAGGTTCATGTCATCCGTC
RpaI_RV1	CAGGTCTCCTCATGAAATCACCTGGAACTCCGG
TAL_FW1	ATTAAAGAGGAGAAAGGTACCATGCTGGCCATGAGCCCGCC
TAL_RV1	TTACACCGGAGATTGTTGCAGC
4Cl_FW1	ATTAAAGAGGAGAAAGGTACCATGGACGCCATGACCGATCCCA
4Cl_RV1	TCACTCCAGCGCAATCACATGC
PchBA_FW1	CAGGTCTCCCATGAAAACTCCCGAAGACTGC
PchBA_RV1	CAGGTCTCCTTAGGCGACGCCGCGCTGCA
Irp9_FW1	CAGGTCTCCATGAAGATCAGCGAGTTCCT
Irp9_RV1	CAGGTCTCCTTAAACCATCAGATACGGCGCG
PhlAC_FW1	GTGAAAGAACGGAATCTGGAGGATGTACAC
PhlAC_RV1	GGTCGGCTCCTTAATCGTTGGA
PhlAC_FW1	TCCAACGATTAAGGAGCCGACC
PhlAC_RV1	TGCTGGTGTTTTATCCGGTCTG
MmfL_FW1	CAGGTCTCCAACCACACCAACCGTCTGCT
MmfL_RV1	CAGGTCTCCTCAGCTCGCAACCGCCTT
MmfH_FW1	CAGGTCTCCACCGCGCCGCTGCGTGAG
MmfH_RV1	CAGGTCTCCTCACGCGGTACGACGTTC
MmfP_FW1	ACTAATAGTACAGGGAGGAGTAATGCCGGAGCCGCCGCGTGAAC
MmfP_RV1	ACACTAGCACTATCAGCGTCAACCGATCGCGCTCGGAT
CHS_FW1	CAGGTCTCCATGGTTACGGTGGAAGAATACCGC
CHS_RV1	CAGGTCTCCTTAGGTAGCCACACTATGCAGAACCA
CHI_FW1	CAGGTCTCCATGGCAGCAAGCATTACGGC
CHI_RV1	CAGGTCTCCTCAGTTACCGATTTTAAAGGCACCTTCA
RhlR_FW1	CAGGTCTCCATGAGGAATGACGGAGGCTT
RhlR_RV1	CAGGTCTCCTTAGATGAGTCCCAGCGCCG
LuxR_FW1	CAGGTCTCCATGAAAAACATAAATGCCGACGAC
LuxR_RV1	CAGGTCTCCTCAATTTTTAAAGTATGGGCAATCAATTGC
CepR_FW1	CAGGTCTCCATGGAACTGCGTTGGCAGGA

CepR_RV1	CAGGTCTCCTTACGGGGCTTCAATCAGACCG
LasR_FW1	CAGGTCTCCATGGCCTTGGTTGACGGTTT
LasR_RV1	CAGGTCTCCTTAGAGAGTAATAAGACCCAAATTAACGGC
BjaR_FW1	CAGGTCTCCATGAGTGCGGTGGATTATGG
BjaR_RV1	CAGGTCTCCTTACGGATTGATAATTTTGTGGCGAA
RpaR_FW1	CAGGTCTCCATGATCGTCGGCGAAGATCAGCT
RpaR_RV1	CAGGTCTCCTCACAAACGGATCAATCCGAGC
NahR_FW1	CAGGTCTCCATGGAACTGCGTGACCTGGAT
NahR_RV1	CAGGTCTCCTCAATCCGTAAACAGGTCAAACA
PhlF_FW1	CAGGTCTCCATGGCACGTACCCCGAGC
PhlF_RV1	CAGGTCTCCTTATACGAATTTTACCCTCGCTTCCACG
MmfR_FW1	CAGGTCTCCATGACCAGCGCGCAGCAA
MmfR_RV1	CAGGTCTCCTTACGCACGCAGCGCAAA
FdeR_FW1	CAGGTCTCCATGCGTTTCAACAAGCTCGA
FdeR_RV1	CAGGTCTCCTCAGCAGATGCCTGGCAG
yIrp9_FW1	CGGATCAATTCGGGATGCATGAAGATCAGCGAGTTCCT
yIrp9_RV1	CGGCCGCTTATTTACTCAGTTAAACCATCAGATACGGCGC
yPchB_FW1	CGGATCAATTCGGGATGCATGAAAACTCCCGAAGACTGC
yPchB_RV1	GCGGCCGCTTATTTACTCAGTCATGCGGCACCCCGTGTCT
yPchA_FW1	GCATACAATCAACTATCTCATATACAATGAGCCGGCTGGCGCCCCTGAGC
yPchA_RV1	TTATTTACTCACGCATGCGACG
VTR3_FW1	ATCCAGCCTCCGCGGCCCCGAATTCATGGGTTCCGGACGGGCTGACGC
VTR3_RV1	AGCTGATCTTCGCCGACGATCCGTCCGGAACCGCTGG
hRpaR_FW1	ATCGTCGGCGAAGATCAGCT
hRpaR_RV1	TCACAAACGGATCAATCCGAGC
h4Cl_FW1	TGCAGGCTGGCGCCACCATGGATGGACGCCATGACCGA
h4Cl_RV1	CTCCAGCGCAATCACATGCG
P2A_FW1	TGTGATTGCGCTGGAGGGATCTGGAGCAACAAACTTCTCACTACT
P2A_RV1	TGCTCACCATGCTACCATCGATTCTAGAAGGCCCGGGATTCTCCTCCACG
hTAL_FW1	GAGGAGAATCCTGGCCCATCTAGAATGCTGGCCATGAGCCCGCC
hTAL_RV1	TTTTGTAATCCAGAGGTTGATTATTTACACCGGAGATTGTTGCAGCA
hRpaI_FW1	GAGGAGAATCCTGGCCCATCTAGAATGCAGGTTCATGTCATCCGTC
hRpaI_RV1	TGAAATCACCTGGAACTCCGGA
iRFP_FW1	TGCAGGCTGGCGCCACCATGGATGGCTGAAGGATCCGTCG
T2A_RV1	TGGGCCAGGATTCTCCTCGA
P2A_FW2	CGGAGTTCCAGGTGATTTCAGGATCTGGAGCAACAAACTTCTCACTACT
P2A_RV2	GGCTCATGGCCAGCATTCTAGAAGGCCCGGGATTCTCCTCCACG

Supplementary Table 4 Primers for amplifying the genes to be expressed in *E. coli* (Green), yeast (Blue) or mammalian (Orange) cells.

Primer Name	Primer Sequence
P_{rhl*}_FW1-2	GAACGATCGTTGGCTGTCCTGTGAAATCTGGCAGTT
P_{rhl*}_RV1-2	GTAAAACTGCCAGATTTCACAGGACAGCCAACGATC
P_{lux*}_FW1-2	GAACGATCGTTGGCTGACCTGTAGGATCGTACAGGT
P_{lux*}_RV1-2	GTAAACCTGTACGATCCTACAGGTCAGCCAACGATC
P_{las*}_FW1-2	GAACGATCGTTGGCTGAACTAGCAAATGAGATAGAT
P_{las*}_RV1-2	GTAAATCTATCTCATTTGCTAGTTCAGCCAACGATC
P_{bja*}_FW1-2	GAACGATCGTTGGCTGTACTGGGAAATTTCCCAATT
P_{bja*}_RV1-2	GTAAAATTGGGAAATTTCCCAGTACAGCCAACGATC
P_{rpa*}_FW1-2	GAACGATCGTTGGCTGACCTGTCCGATCGGACAGTT
P_{rpa*}_RV1-2	GTAAAACTGTCCGATCGGACAGGTCAGCCAACGATC
P_{mut*}_FW1-3	TTACGCAAGAAAATGGTTTGTTACTTTCGAATAAA
P_{mut*}_RV1-3	AGCTTTTATTCGAAAGTAACAAACCATTTTCTTGC
P_{cep*}_FW1-2	TGATCGGAACGATCGTTGGCTGCGCCGTCACC
P_{cep*}_RV1-2	ACAGGGTGACGGCGCAGCCAACGATCGTTCCG
P_{cep*}_FW1-3	CTGTAAGAGTTACCAGTTTACGCAA
P_{cep*}_RV1-3	TTTCTTGCGTAAACTGGTAACTCTT
P_{cep*}_FW1-4	GAAAATGGTTTGTTACTTTCGAATAAA
P_{cep*}_RV1-4	AGCTTTTATTCGAAAGTAACAAACCAT
P_{phlF}_FW1-2	AGCGGGTAAAGTAACTATGATTCGTTACCAATTGAC
P_{phlF}_FW1-2	TCATGTCAATTGGTAACGAATCATAGTTACTTTACC
P_{phlF}_FW1-3	ATGATACGAAACGTACCGTATCGTTAAGGTTACTAGAG
P_{phlF}_FW1-3	AGCTCTCTAGTAACCTTAACGATACGGTACGTTTCGTA
P_{mmf}_FW1-2	TCTGATCGGAACGATCGTTGGCTGTT
P_{mmf}_RV1-2	TGTCAACAGCCAACGATCGTTCCGAT
P_{mmf}_FW1-3	GACAAACCTTCGGGAAGGTATGATACTCGAATAG
P_{mmf}_RV1-3	AGCTCTATTCGAGTATCATACCTTCCCGAAGGTT
P_{sal}_FW1	CAGGTCTCCCCTGGGGGCCTCGCTTGGGTTATT
P_{sal}_RV1	CAGGTCTCCAGCTGATCCTCTATGGTACTCGTGATGG
P_{fdeA}_FW1	CAGGTCTCACCTGTTGTGCTTGTTCTTGCCG
P_{fdeA}_RV1	GTGGTCTCCAGCTCTCCTTGTCTCCTACCCTGC
P_{gal}_FW1	CCCTTTAGTGAGGGTTGAATTCGAAT
P_{gal}_RV1	CCGGGGTTTTTTCTCCTTGACG
$P_{rpaO\text{-}CMV}$_FW1-1	TTCAGGTACCACCTGTCCGATCGGACAGTT
$P_{rpaO\text{-}CMV}$_RV1-1	TATCAACTGTCCGATCGGACAGGTGGTACC
$P_{rpaO\text{-}CMV}$_FW1-2	GATATCACCTGTCCGATCGGACAGTTCTCGAGAGC
$P_{rpaO\text{-}CMV}$_RV1-2	CGAGGCTCTCGAGAACTGTCCGATCGGACAGGTGA
$P_{rpaO\text{-}CMV}$_RV2-2	CCGAGCTCTCGAGAACTGTCCGATCGGACAGGTGA
CMV1_FW1-1	CAGGTCTCCTCGGTACCCGGGTCGAGGTAGGCGTGTACGG
CMV1_FW2-1	AGGTAGGCGTGTACGGTGGGAGGCCTATATAAGCAGAGCTCGTTTAGT
CMV1_RV1-1	CAGGTCTCCAGGCGATCTGACGGTTCACTAAACGAGCTCTGCTTATATAG
CMV1_FW1-2	CAGGTCTCCGCCTGGAGACGCCATCCACGCTGTTTTGACC

CMV1_FW2-2	CCACGCTGTTTTGACCTCCATAGAAGACACCGGGACCGATCCAG
CMV1_RV1-2	CAGGTCTCCAGCTGCCGCGGAGGCTGGATCGGTCCCGGT
TRE3G_FW1	CAGGTCTCCCTCGGTACCCGGGTCGAGGTAGGCGTGTACGGTGGGC
TRE3G_RV1	CAGGTCTCCAGCTTTTACGAGGGTAGGAAGTGG

Supplementary Table 5 Primers for constructing or amplifying the signal responding promoters in *E. coli* (Green), yeast (Blue) or mammalian (Orange) receivers.

a

GFP	RhlR + P_{rhl*}	LuxR + P_{lux*}	CepR + P_{cep*}	LasR + P_{las*}	BjaR + P_{bja*}	RpaR + P_{rpa*}	NahR + P_{sal}	PhlF + P_{phlF}	MmfR + P_{mmfR}	FdeR + P_{fdeA}
C4	29348	2335	2577	156	3711	1475	554	184	102	108
3OC6	2267	4217	921	289	147	352	443	183	37	110
C8	5401	2847	4968	839	750	494	437	196	243	95
3OC12	589	93	838	1367	68	498	517	222	181	318
IV	501	44	84	59	1606	458	471	168	62	48
pC	558	66	481	90	66	32512	486	181	98	113
Sal	368	89	123	97	79	452	6238	299	132	119
DAPG	422	47	85	57	53	400	481	19296	65	127
MMF	530	38	57	54	47	562	67	105	1008	106
NG	606	41	69	60	54	721	90	106	94	752
NO induction	350	59	52	50	35	225	323	55	55	58

b

Fold-change	RhlR + P_{rhl*}	LuxR + P_{lux*}	CepR + P_{cep*}	LasR + P_{las*}	BjaR + P_{bja*}	RpaR + P_{rpa*}	NahR + P_{sal}	PhlF + P_{phlF}	MmfR + P_{mmfR}	FdeR + P_{fdeA}
C4	83.81	39.53	49.75	3.13	106.54	6.55	1.72	3.34	1.85	1.86
3OC6	6.47	71.37	17.78	5.78	4.22	1.56	1.37	3.32	0.66	1.90
C8	15.42	48.19	95.91	16.80	21.54	2.19	1.35	3.55	4.39	1.63
3OC12	1.68	1.57	16.19	27.36	1.95	2.21	1.60	4.03	3.27	5.48
IV	1.43	0.74	1.62	1.19	46.11	2.04	1.46	3.04	1.11	0.83
pC	1.59	1.12	9.28	1.80	1.89	144.41	1.50	3.29	1.78	1.94
Sal	1.05	1.51	2.37	1.94	2.27	2.01	19.32	5.42	2.39	2.05
DAPG	1.20	0.79	1.64	1.13	1.54	1.78	1.49	350.12	1.17	2.19
MMF	1.51	0.64	1.11	1.07	1.36	2.49	0.21	1.90	18.23	1.83
NG	1.73	0.69	1.33	1.21	1.54	3.20	0.28	1.92	1.69	12.97

Supplementary Table 6 Data processing of signal orthogonality. First column indicates the inducers in sender media. First row indicates the allosteric TFs and their responding promoters. Shaded cells indicate the fluorescence intensity or fold-change of receivers activated by cognate sender media. **a** Mean fluorescence of each sample. Red numbers in the bottom row indicate background fluorescence before induction. **b** Fold-change of each sample. Fluorescence intensity of each sample in (a) was divided by the background intensity in the same column. Data represent the mean of fluorescence of three replicates.

a

aTF	Inducer	P_{rhl*}	P_{lux*}	P_{cep*}	P_{las*}	P_{bja*}	P_{rpa*}	P_{sal}	P_{phlF}	P_{mmfR}	P_{fdeA}
RhlR	+ C4	20478	777	2831	11703	173	2138	339	28746	18343	171
	- C4	279	18	30	183	46	148	413	32859	15476	346
LuxR	+ 3OC6	17026	3759	9187	26	91	9108	445	32877	24391	256
	- 3OC6	255	19	24	30	49	66	432	31899	22302	254
CepR	+ C8	10914	1052	2525	27	5528	5163	498	32277	24026	290
	- C8	267	21	25	28	18	63	417	31083	22539	223
LasR	+ 3OC12	157	194	1523	2147	14290	2885	295	20395	6772	69
	- 3OC12	223	22	25	49	1270	364	390	30466	19744	256
BjaR	+ IV	266	18	20	22	3478	54	573	18319	24179	316
	- IV	273	15	15	23	14	54	486	20198	20352	285
RpaR	+ pC	377	1308	31	24	132	17231	438	32576	24219	294
	- pC	270	17	13	22	78	155	400	32823	23377	307
NahR	+ Sal	160	14	15	21	25	27	3462	30282	22850	263
	- Sal	267	16	16	22	30	39	246	32996	20757	238
PhlF-	+ DAPG	232	14	21	22	13	40	417	17050	24306	273
	- DAPG	261	15	22	21	13	20	429	167	22014	248
MmfR	+ MMF	297	13	16	19	56	14	201	19318	912	131
	- MMF	186	26	30	40	29	27	218	25340	29	149
FdeR	+ NG	262	22	21	33	24	26	303	29654	18199	10954
	- NG	233	19	20	28	21	24	331	28779	21351	74

b

Fold-change	P_{rhl*}	P_{lux*}	P_{cep*}	P_{las*}	P_{bja*}	P_{rpa*}	P_{sal}	P_{phlF}	P_{mmfR}	P_{fdeA}
C4 + RhlR	73.53	42.55	95.27	63.88	3.73	14.41	0.82	0.87	1.19	0.49
3OC6 + LuxR	66.73	198.52	377.81	0.86	1.87	137.47	1.03	1.03	1.09	1.01
C8 + CepR	40.90	49.34	101.69	0.96	312.32	82.09	1.19	1.04	1.07	1.30
3OC12 + LasR	0.71	8.92	60.26	43.56	11.25	7.92	0.76	0.67	0.34	0.27
IV + BjaR	0.97	1.21	1.31	0.95	241.23	1.00	1.18	0.91	1.19	1.11
pC + RpaR	1.40	79.04	2.46	1.06	1.70	111.05	1.09	0.99	1.04	0.96
Sal + NahR	0.60	0.91	0.92	0.95	0.83	0.70	14.10	0.92	1.10	1.10
DAPG + PhlF	0.89	0.95	0.94	1.01	1.07	1.99	0.97	102.08	1.10	1.10
MMF + MmfR	1.60	0.50	0.55	0.46	1.92	0.52	0.92	0.76	31.06	0.88
NG + FdeR	1.12	1.14	1.04	1.17	1.10	1.10	0.92	1.03	0.85	147.78

Supplementary Table 7 Data processing of promoter orthogonality. First row indicates the allosteric TF responding promoters. Shaded cells indicate the fluorescence intensity or fold-change of promoters activated by cognate aTFs. **a** Mean fluorescence of each sample. First and second columns indicate the aTFs and their corresponding inducers, respectively. Red numbers indicate background fluorescence level without induction of signa molecules. **b** Fold-change of each sample. Fluorescence intensity of each sample (with signal molecule) in (a) was divided by the background intensity (without signal molecule). Data represent the mean fluorescence of three replicates.

Supplementary References

1. Lindemann A, *et al.* Isovaleryl-homoserine lactone, an unusual branched-chain quorum-sensing signal from the soybean symbiont Bradyrhizobium japonicum. *Proceedings of the National Academy of Sciences of the United States of America* **108**, 16765-16770 (2011).

2. Hirakawa H, *et al.* Activity of the Rhodopseudomonas palustris p-coumaroyl-homoserine lactone-responsive transcription factor RpaR. *J Bacteriol* **193**, 2598-2607 (2011).

3. O'Rourke S, Wietzorrek A, Fowler K, Corre C, Challis GL, Chater KF. Extracellular signalling, translational control, two repressors and an activator all contribute to the regulation of methylenomycin production in Streptomyces coelicolor. *Mol Microbiol* **71**, 763-778 (2009).

4. Wei Y, Ryan GT, Flores-Mireles AL, Costa ED, Schneider DJ, Winans SC. Saturation mutagenesis of a CepR binding site as a means to identify new quorum-regulated promoters in Burkholderia cenocepacia. *Mol Microbiol* **79**, 616-632 (2011).

5. Moon TS, Lou C, Tamsir A, Stanton BC, Voigt CA. Genetic programs constructed from layered logic gates in single cells. *Nature* **491**, 249-253 (2012).

6. Chuang JS, Rivoire O, Leibler S. Simpson's paradox in a synthetic microbial system. *Science* **323**, 272-275 (2009).

7. Xue H, *et al.* Design, construction, and characterization of a set of biosensors for aromatic compounds. *Acs Synth Biol* **3**, 1011-1014 (2014).

8. Stanton BC, Nielsen AA, Tamsir A, Clancy K, Peterson T, Voigt CA. Genomic mining of prokaryotic repressors for orthogonal logic gates. *Nat Chem Biol* **10**, 99-105 (2014).

9. Hou J, *et al.* Engineering the Ultrasensitive Transcription Factors by Fusing a Modular Oligomerization Domain. *Acs Synth Biol*, (2018).

10. Siedler S, Stahlhut SG, Malla S, Maury J, Neves AR. Novel biosensors based on flavonoid-responsive transcriptional regulators introduced into Escherichia coli. *Metabolic engineering* **21**, 2-8 (2014).

11. Wu J, Zhou T, Du G, Zhou J, Chen J. Modular optimization of heterologous pathways for de novo synthesis of (2S)-naringenin in Escherichia coli. *Plos One* **9**, e101492 (2014).

12. Brachmann AO, *et al.* Pyrones as bacterial signaling molecules. *Nat Chem Biol* **9**, 573-U573 (2013).

13. Liang C, Xiong D, Zhang Y, Mu S, Tang SY. Development of a novel uric-acid-responsive regulatory system in Escherichia coli. *Applied microbiology and biotechnology* **99**, 2267-2275 (2015).

14. Ohnishi Y, Kameyama S, Onaka H, Horinouchi S. The A-factor regulatory cascade leading to streptomycin biosynthesis in Streptomyces griseus : identification of a target gene of the A-factor receptor. *Mol Microbiol* **34**, 102-111 (1999).

15. Takano E, *et al.* A bacterial hormone (the SCB1) directly controls the expression of a pathway-specific regulatory gene in the cryptic type I polyketide biosynthetic gene cluster of Streptomyces coelicolor. *Mol Microbiol* **56**, 465-479 (2005).

16. Ma D, Peng S, Huang W, Cai Z, Xie Z. Rational Design of Mini-Cas9 for Transcriptional Activation. *Acs Synth Biol* **7**, 978-985 (2018).

17. Sedlmayer F, Jaeger T, Jenal U, Fussenegger M. Quorum-Quenching Human Designer Cells for Closed-Loop Control of Pseudomonas aeruginosa Biofilms. *Nano Lett* **17**, 5043-5050 (2017).

18. Bangera MG, Thomashow LS. Identification and characterization of a gene cluster for synthesis of the polyketide antibiotic 2,4-diacetylphloroglucinol from Pseudomonas fluorescens Q2-87. *J Bacteriol* **181**, 3155-3163 (1999).

19. Thomashow LS. Biological control of plant root pathogens. *Curr Opin Biotechnol* **7**, 343-347 (1996).

20. Price CT, Lee IR, Gustafson JE. The effects of salicylate on bacteria. *Int J Biochem Cell Biol* **32**, 1029-1043 (2000).
