# Supplementary material for: De novo design of an intercellular signaling toolbox for multi-channel cell–cell communication and biological computation
Source: Nat Commun. 2020 Aug 24;11:4226. doi: 10.1038/s41467-020-17993-w (PMC7445162; doi:10.1038/s41467-020-17993-w)
Supplement: Supplementary file 3 — Reporting Summary [file 41467_2020_17993_MOESM3_ESM.pdf]

nature research

Corresponding author(s): Chunbo Lou
Last updated by author(s): Jul 15, 2020

Reporting Summary

Nature Research wishes to improve the reproducibility of the work that we publish. This form provides structure for consistency and transparency in reporting. For further information on Nature Research policies, see our Editorial Policies and the Editorial Policy Checklist.

Statistics

For all statistical analyses, confirm that the following items are present in the figure legend, table legend, main text, or Methods section.

n/a	Confirmed	
☐	☒	The exact sample size (*n*) for each experimental group/condition, given as a discrete number and unit of measurement
☐	☒	A statement on whether measurements were taken from distinct samples or whether the same sample was measured repeatedly
☒	☐	The statistical test(s) used AND whether they are one- or two-sided<br>*Only common tests should be described solely by name; describe more complex techniques in the Methods section.*
☒	☐	A description of all covariates tested
☒	☐	A description of any assumptions or corrections, such as tests of normality and adjustment for multiple comparisons
☐	☒	A full description of the statistical parameters including central tendency (e.g. means) or other basic estimates (e.g. regression coefficient) AND variation (e.g. standard deviation) or associated estimates of uncertainty (e.g. confidence intervals)
☒	☐	For null hypothesis testing, the test statistic (e.g. *F*, *t*, *r*) with confidence intervals, effect sizes, degrees of freedom and *P* value noted<br>*Give P values as exact values whenever suitable.*
☒	☐	For Bayesian analysis, information on the choice of priors and Markov chain Monte Carlo settings
☒	☐	For hierarchical and complex designs, identification of the appropriate level for tests and full reporting of outcomes
☒	☐	Estimates of effect sizes (e.g. Cohen's *d*, Pearson's *r*), indicating how they were calculated

Our web collection on statistics for biologists contains articles on many of the points above.

Software and code

Policy information about availability of computer code

Data collection	No commercial, open source or custom code was used to collect data in this study.
Data analysis	Matlab code for analyzing mammalian flow cytometry data with internal control is available at git@github.com:xjpatriot87/Mammalian-data-analysis-with-matlab.git

For manuscripts utilizing custom algorithms or software that are central to the research but not yet described in published literature, software must be made available to editors and reviewers. We strongly encourage code deposition in a community repository (e.g. GitHub). See the Nature Research guidelines for submitting code & software for further information.

Data

Policy information about availability of data

All manuscripts must include a data availability statement. This statement should provide the following information, where applicable:

- Accession codes, unique identifiers, or web links for publicly available datasets
- A list of figures that have associated raw data
- A description of any restrictions on data availability

We declare that all relevant data supporting the findings of this study are available within the article and its Supplementary Information Files or from the corresponding authors upon request.

Field-specific reporting

Please select the one below that is the best fit for your research. If you are not sure, read the appropriate sections before making your selection.

- [x] Life sciences
- [ ] Behavioural & social sciences
- [ ] Ecological, evolutionary & environmental sciences

For a reference copy of the document with all sections, see nature.com/documents/nr-reporting-summary-flat.pdf

Life sciences study design

All studies must disclose on these points even when the disclosure is negative.

Sample size	All samples included at least 3 replicates to generate mean and standard deviation.
Data exclusions	No data were excluded from the analysis.
Replication	All attempts at replication were successful.
Randomization	Randomization is not relevant in this study.
Blinding	Blinding is not relevant in this study. No experiment required blinding of investigator.

Reporting for specific materials, systems and methods

We require information from authors about some types of materials, experimental systems and methods used in many studies. Here, indicate whether each material, system or method listed is relevant to your study. If you are not sure if a list item applies to your research, read the appropriate section before selecting a response.

Materials & experimental systems

n/a	Involved in the study	
x		Antibodies
	x	Eukaryotic cell lines
x		Palaeontology and archaeology
x		Animals and other organisms
x		Human research participants
x		Clinical data
x		Dual use research of concern

Methods

n/a	Involved in the study	
x		ChIP-seq
	x	Flow cytometry
x		MRI-based neuroimaging

Eukaryotic cell lines

Policy information about cell lines

Cell line source(s)	The human kidney epithelial cell line HEK293T (ATCC CRL-11268)
Authentication	The morphology of HEK293T cell-line were visually authenticated with microscope before use.
Mycoplasma contamination	All cell line were test negative for mycoplasma contamination.
Commonly misidentified lines (See ICLAC register)	No cell line is commonly misidentified with HEK293T.

Flow Cytometry

Plots

Confirm that:

- [x] The axis labels state the marker and fluorochrome used (e.g. CD4-FITC).
- [x] The axis scales are clearly visible. Include numbers along axes only for bottom left plot of group (a 'group' is an analysis of identical markers).
- [x] All plots are contour plots with outliers or pseudocolor plots.
- [ ] A numerical value for number of cells or percentage (with statistics) is provided.

Methodology

Sample preparation	E. coli, yeast or HEK293T cells were cultured and induced with methods indicated in the Material & Method section before flow cytometry. No tissue is involved in this study.
Instrument	BD LSRII for all flow cytometry data collection, BD BD FACSAria IIIu for sorting of HEK293T cells.
Software	The mean fluorescence of each sample was analyzed using FlowJo X (TreeStar Inc., Ashland, OR) except the data of HEK293T cell. The fluorescent data of HEK293T cells were analyzed using Matlab 2018b (The MathWorks Inc., Natick, MA). The matlab code is deposited in GitHub (git@github.com:xjpatriot87/Mammalian-data-analysis-with-matlab.git)
Cell population abundance	All flow cytometry data include 10000-100000 cells as indicated by the total events collected by flow cytometer. HEK293T cells were sorted for double-positive (Alexa Fluor 700 and BV421) population.
Gating strategy	No gating was applied for all flow cytometry data collection.

☐ Tick this box to confirm that a figure exemplifying the gating strategy is provided in the Supplementary Information.
