# Supplementary material for: De novo design of an intercellular signaling toolbox for multi-channel cell–cell communication and biological computation
Source: Nat Commun. 2020 Aug 24;11:4226. doi: 10.1038/s41467-020-17993-w (PMC7445162; doi:10.1038/s41467-020-17993-w)
Supplement: Supplementary file 4 — Description of Additional Supplementary Files [file 41467_2020_17993_MOESM4_ESM.pdf]

Title: Supplementary Data 1
Description: Sequences of the promoter-gene-terminator cassettes in the *E. coli*, yeast and mammalian senders and receivers. $T_{adh1\text{-}C}$ and $T_{adh1\text{-}Y}$ indicate the adh1 terminator from *C.albicans* and *S. cerevisiae*, respectively. The J23101-*tetR-T* and P_{lacI}-*lacI-T* cassettes are included where P_{tet} and P_{tac} promoters are used, respectively. The J23119-*mRFP1* cassette is expressed in all *E. coli* senders to label sender cells for co-culture. The riboJ-*sfGFP-T* cassette is inserted downstream of each promoter shown in Supplementary Table 2. The CMV1 or CMV3G promoter is inserted upstream of the *Citrine*-rbGlob_p(A)-$P_{hEF1\alpha}$-*VTR3-rpaR*-hGH_p(A)-P_{EF1}-*mCherry*-SV40_p(A) cassette.
